# Artificial intelligence-based optimization for chitosan nanoparticles biosynthesis, characterization and in‑vitro assessment of its anti-biofilm potentiality

**DOI:** 10.1038/s41598-023-30911-6

**Published:** 2023-03-16

**Authors:** Noura El-Ahmady El-Naggar, Shimaa R. Dalal, Amal M. Zweil, Marwa Eltarahony

**Affiliations:** 1 Department of Bioprocess Development, Genetic Engineering and Biotechnology Research Institute, City of Scientific Research and Technological Applications (SRTA-City), New Borg El-Arab City, 21934 Alexandria Egypt; 2 Botany Department, Faculty of Science, Mansoura University, Mansoura, Egypt; 3 Plant Biotechnology Department, Genetic Engineering and Biotechnology Research Institute, University of Sadat City, Sadat City, Egypt; 4 Environmental Biotechnology Department, Genetic Engineering and Biotechnology Research Institute (GEBRI), City of Scientific Research and Technological Applications (SRTA-City), New Borg El-Arab City, 21934 Alexandria Egypt

**Keywords:** Nanoparticles, Nanoparticles

## Abstract

Chitosan nanoparticles (CNPs) are promising biopolymeric nanoparticles with excellent physicochemical, antimicrobial, and biological properties. CNPs have a wide range of applications due to their unique characteristics, including plant growth promotion and protection, drug delivery, antimicrobials, and encapsulation. The current study describes an alternative, biologically-based strategy for CNPs biosynthesis using *Olea* *europaea* leaves extract. Face centered central composite design (FCCCD), with 50 experiments was used for optimization of CNPs biosynthesis. The artificial neural network (ANN) was employed for analyzing, validating, and predicting CNPs biosynthesis using *Olea* *europaea* leaves extract. Using the desirability function, the optimum conditions for maximum CNPs biosynthesis were determined theoretically and verified experimentally. The highest experimental yield of CNPs (21.15 mg CNPs/mL) was obtained using chitosan solution of 1%, leaves extract solution of 100%, initial pH 4.47, and incubation time of 60 min at 53.83°C. The SEM and TEM images revealed that CNPs had a spherical form and varied in size between 6.91 and 11.14 nm. X-ray diffraction demonstrates the crystalline nature of CNPs. The surface of the CNPs is positively charged, having a Zeta potential of 33.1 mV. FTIR analysis revealed various functional groups including C–H, C–O, CONH_2_, NH_2_, C–OH and C–O–C. The thermogravimetric investigation indicated that CNPs are thermally stable. The CNPs were able to suppress biofilm formation by *P.* *aeruginosa,* *S.* *aureus* and *C.* *albicans* at concentrations ranging from 10 to 1500 µg/mL in a dose-dependent manner. Inhibition of biofilm formation was associated with suppression of metabolic activity, protein/exopolysaccharide moieties, and hydrophobicity of biofilm encased cells (r ˃ 0.9, *P* = 0.00). Due to their small size, in the range of 6.91 to 11.14 nm, CNPs produced using *Olea* *europaea* leaves extract are promising for applications in the medical and pharmaceutical industries, in addition to their potential application in controlling multidrug-resistant microorganisms, especially those associated with post COVID-19 pneumonia in immunosuppressed patients.

## Introduction

Green nanotechnology includes the biological synthesis of nanoparticles using microorganisms, plants or their products, e.g., lipids and proteins^1^. Nanoparticle biosynthesis is primarily limited to metal ions, however, the number of studies on the bioconversion of organic compounds into nanoparticles is limited. Polymeric nanoparticles are organic based nanoparticles that can be synthesized from natural and synthetic polymers. Some of the merits of biopolymeric nanoparticles include availability of marine or agricultural resources, biodegradability, biocompatibility and non-toxicity^2^.

Chitosan is a biodegradable linear polysaccharide produced from the chitin biopolymer via partial deacetylation. It is a nitrogenous, white, rigid polysaccharide^3^. It is composed of alternate units of *N*-acetyl glucosamine and deacetylated units of glucosamine linked by β-(1 → 4) glycosidic bonds. Chitosan possesses a variety of useful characteristics, including its polycationic nature, non-toxicity, biodegradability, biocompatibility, and antibacterial capabilities. Chitosan is used extensively in the biomedical industry, paper manufacture, for the encapsulation of active food ingredients, water treatment, environmental pollution control, photography, in agriculture as a plant growth promoter, as a carrier for controlled drug delivery, and enzyme immobilization^4^.

Although, bulk chitosan is completely environmentally friendly and renewable, its poor solubility in aqueous media limits its antimicrobial activity^5^. Chitosan has been used in the fabrication of nanoparticles^6^. When compared to chitosan in its bulk form, nanoparticles are much easier for cells to internalize. The reduction in size and dimension of nanoparticles results in an increase in the surface area to volume ratio, as well as an increase in the surface area in contact with microbes. These nanoparticles can also accumulate at the target sites of the pathogenic microorganisms and interact with the negatively charged surface of the bacterial cell^7^. Therefore, the bioavailability, efficiency, and therapeutic stability are improved in comparison to the same amount of bulk chitosan particles^1^. CNPs possess the characteristics of both chitosan and nanoparticles, including quantum size effects, small size, and the surface and interface effect^7^.

CNPs have promising potential for various applications, such as controlled and continuous drug release^8^, they can be an asset in drug delivery by reducing the damage to non-targeted tissue or cells^9^, or by preventing the enzymatic degradation of drugs^10^, for cancer treatment and for biological imaging and diagnosis^11^. CNPs can also be used for herbicide delivery for weed eradication^12^, as a nanofertilizer for delivering balanced nutrition to plants^13^, in sustainable agriculture as a carrier for the plant growth hormone gibberellic acid^14^, in insecticide treatment^15^ and fungicide treatment^16^.

Antimicrobial multidrug resistance (MDR) has progressively developed over the past few decades, and is one of the most critical concerns in human health, since many infectious organisms are becoming more resistant to currently marketed antimicrobial medications^17–19^. In a natural environment, microorganisms rarely exist in a planktonic mode and gravitate irreversibly towards the air/liquid, liquid/liquid, and air/solid interfacial surfaces in the form of three-dimensional complex aggregates (i.e., multicellular communities) or biofilms. Via such lifestyle, they immobilize themselves in a hydrated, sticky self-protecting barrier of extracellular polymeric matrix (EPS), which is composed of extracellular polysaccharides (EPS), proteins, lipids, phospholipids, teichoic acids, surfactants, humic substances and eDNA^20,21^. Chronic infections, including those related to medical devices, can be caused by MDR bacteria growing within biofilm. Biofilms have been found in numerous medical devices such as prosthetic devices, urinary catheters, dental irrigation units and dental plaques, pacemakers, colonoscopes, contact lenses, endoscopes^22^. In order to eradicate biofilms and reduce the risk that they posed, a number of different mechanical removal strategies and chemical biocides are utilized^23^. The pharmaceutical industry is in critical need to exploring new drugs due to the rise in frequency of pathogenic multidrug-resistant microorganisms^24^. Chitosan nanoparticles exhibit strong antimicrobial action against pathogens like, *Candida* *albicans*, *Escherichia* *coli*, *Staphylococcus* *aureus*, *Pseudomonas* *aeruginosa* and *Klebsiella* *pneumoniae*^4,25^. Recently, the nanocomposites formulations of chitosan impregnated with other metal nanoparticles (Ag, ZnO, etc.) have been able to improve its biological activities^26^. Gingasu et al.^27^ found that coating chitosan with cobalt ferrites nanoparticles enhanced its antibacterial activity; reflecting mutual enforcement in defeating microbial pathogens either in planktonic or sessile lifestyles. Similarly, combining chitosan with other natural materials such as citronella essential oil exhibited a prominent antimicrobial potency; revealing the possibility of applying such innovative films of chitosan-based materials as antimicrobial coating agents in food packaging for preservation and extension of food shelf-life^28^.

Chitosan nanoparticles can be prepared by various methods, including the ionic gelation method^29^, ionic gelation with radical polymerization self-assembly, precipitation-based methods, emulsification & crosslinking, spray drying, and supercritical-CO_2_-assisted solubilization and atomization^30^. Chemical and physical techniques have numerous drawbacks, including the utilization of high pressure, energy, temperature, hazardous compounds, and can result in large particle size^30^. Consequently, there is an urgent need to establish environmentally-friendly technologies for the ultrafine production of CNPs. Shankar et al.^31^ reported that various organisms, including bacteria, fungi, and plants, have been used for the biosynthesis and assembly of nanoparticles. During the process of nanoparticle biosynthesis, it is hypothesized that biological agents participate either as stabilizers, reducers, or both^33^.The production of ultrafine CNPs with a size of less than 100 nm, which is an essential quality for a great number of applications, where specific surface area is an important limiting factor, was accomplished through the use of environmentally friendly method^32^.

Optimization of the biosynthesis process for chitosan nanoparticles can be achieved using the traditional technique by modifying a single variable while maintaining other variables at their optimal levels. Application of this traditional technique has some drawbacks, such as being tedious, hard, prolonged, consumption of reagents and materials, in order to determine optimal conditions. Moreover, the traditional technique ignores interaction effects among the independent factors^34^. A Face centered central composite design (FCCCD) had been previously used to assess the effects of processing factors on biosynthesis of CNPs. It is one of the statistical and mathematical techniques that could be applied to optimize the variables at the same time, which is faster, more cost effective, minimizes the sum of experimental trials for multiple parameters, is applicable, defines the best conditions, and keeps high accuracy of the final outcome in comparison with the traditional technique^35–37^. Currently, artificial neural networks (ANNs) are the most widely used artificial learning technique in biotechnology. ANNs have a wide range of applications, including the optimization of bioprocesses^38^.

The present work describes a simple, cost-effective, and eco-friendly technique for chitosan nanoparticles biosynthesis as well as the characterization of the obtained nanoparticles. Face centered central composite design (FCCCD), with 50 experiments, was performed to optimize the process of chitosan nanoparticles biosynthesis. Finally, evaluation of their inhibitory activity against microbial biofilm was performed.

## Materials and methods

### Plant-extract preparation

Fresh *Olea* *europaea* leaves were gained from region of Wadi El Natrun, Northern West Nile Delta of Egypt, 62 miles from Cairo (at a latitude of 30°22′39′′ North and a longitude of 30°21′1.08′′ East). The plant was kindly identified by Prof. Dr. Nazmy Abdel Hamid Abdel-Ghany, Horticulture Department, Faculty of Agriculture, Ain Shams University, Cairo, Egypt. The voucher specimen (*Olea* *europaea*) has been deposited at the herbarium of Environmental Studies and Research Institute, at University of Sadat City, Egypt. “The *Olea* *europaea* leaves were collected, with permission, according to institutional, national, and international guidelines and legislation”. After being washed thoroughly with tap water three times, the leaves were given a final rinse with distilled water to remove any remaining contaminants. 25 g of well-washed leaves of *Olea* *europaea* were chopped into small pieces into a conical flask containing 100 mL of distilled water, immersed, stirred, and boiled for 10 min. The boiled solution of the *Olea* *europaea* leaves was filtered with filter paper (Whatman No. 1) and the resultant filtered extract was collected and used in the CNPs biosynthesis.

### Green synthesis of chitosan nanoparticles

Chitosan was obtained from Bio Basic Inc., Toronto, Canada, with purity > 90% and viscosity 60–300). Chitosan (1%, w/v) was liquefied with acetic acid at a concentration of 1 percent (v/v). The pH was adjusted to 4.8 ± 0.02 with 1N NaOH and kept under magnetic stirring for 24 h to ensure that the chitosan was entirely dissolved. Equal amounts (10 mL) from each of *Olea* *europaea* leaves extract and chitosan solution were mixed and incubated at 50 °C for 50 min while being shaken at 110 rpm. After incubation, the turbid mixture was subjected to centrifugation at 10,000×*g* for ten minutes, and the resulting pellets washed, and then freeze-dried. A double beam spectrophotometer was used to analyze the UV/VIS absorbance spectrum of the biosynthesized CNPs. To quantify the final concentrations of CNPs (mg/mL), known concentration of CNPs was prepared in 1% acetic and subjected to serial dilutions to generate a standard calibration curve.

## Characterization of CNPs

### UV–visible spectrum

To determine the maximum absorbance wavelength, the biosynthesized CNPs were analyzed by scanning at a wavelength range between 200 and 400 nm using an Optizen Pop-UV/Vis spectrophotometer.

### SEM and TEM investigations of CNPs samples

Chitosan nanoparticles size and morphology were examined by JEOL-JSM-IT200 Scan Electron Microscope at the Electron Microscope Unit, Faculty of Science, Alexandria University, Alexandria, Egypt. Transmission Electron Microscope (TEM) investigation was performed using TEM (JEOL-JEM-2100 Plus, Ltd., Japan) at the Central Laboratory, City of Scientific Research and Technological Applications, Alexandria, Egypt. The elemental composition of a sample can be determined using Energy Dispersive X-ray (EDX) spectroscopy analysis using TEM. Mapping analysis was performed using TEM to demonstrate the composition and distribution of chitosan nanoparticles.

### Zeta-potential of the synthesized CNPs

Zeta potential is used extensively for determination of the surface charge and nanoparticles stability^39^. The ζ-potential of the chitosan nanoparticles was quantified utilizing a Malvern 3000 Zetasizer Nano ZS, UK at "Central Laboratories, City of Scientific Research and Technological Applications, Alexandria, Egypt". Before the analysis, the suspension of diluted CNPs was first homogenized in a high-speed homogenizer at 13,000 rpm for 10 min, after which it was maintained in an ultrasonic bath. The sample was tested three times.

### FTIR spectroscopy analysis

FTIR spectroscopy was used to analyze the surface characteristics of the chitosan nanoparticles. Shimadzu FTIR-8400 S spectrophotometer was used to measure the FTIR spectrum of CNPs, with a resolution of 1 cm^−1^ and a range of 4500–500 cm^−1^.

### XRD pattern

The crystallinity of the CNPs and structural properties were determined by XRD using advanced X-ray diffractometer (Bruker D2 Phaser 2nd Gen) equipped with a CuKα radiation, λ = 1.5406 Å source (applied voltage 10 kV, current 30 mA). Data was obtained at a scanning rate of 2°/min for 2θ between 10 and 60.

### CNPs' thermal characteristics

The pyrolysis pattern of chitosan nanoparticles was examined using a Differential Scanning Calorimetry (DSC) analysis. Freeze-drying CNPs sample of approximately 3.2 mg was analyzed at a flow rate of 30 mL/min under nitrogen atmosphere conditions. The scan temperatures ranging from ambient temperature to 500 °C.

Thermogravimetric analysis (TGA) of CNPs analysis was attained using a thermo-analyzer of type 50-H. For TGA analysis, approximately 6 mg of the sample were subjected to temperatures ranging from ambient temperature to 800 °C at an increment of 10 °C min^−1^. The sample was investigated at a 40 mL/min flow rate.

### Optimization of CNPs by FCCCD

Optimum levels of five independent variables and their effects on CNPs biosynthesis were determined by FCCCD. The independent variables tested were: chitosan concentration (%) (X_1_; 0.5, 1, 1.5), concentration of leaves extract (%) (X_2_; 50, 75, 100%), initial pH level (X_3_; 4, 4.5, 5), temperature (ºC) (X_4_; 40, 50, 60) and incubation time (min.) (X_5_; 60, 90, 120). The experimental design used in this study consisted of 50 experimental trials including 8 trials at the central point. The theoretical relationships among the independent variables and the outcomes (CNPs biosynthesis, mg/mL) were identified by applying the polynomial equation of the second degree.

1 $${\text{Y }} = \beta_{0} + \sum\limits_{i} {\beta_{i} X_{i} } + \sum\limits_{ii} {\beta_{ii} X_{i}^{{2}} } + \sum\limits_{ij} {\beta_{ij} X_{i} X_{j} }$$

In which Y is the predicted (CNPs biosynthesis, mg/mL), the linear coefficient (β_i_), quadratic coefficients (β_ii_), (β_0_) represents the regression coefficients, (β_ij_) the interaction coefficients and (X_i_) is the coded values of the independent factors.

### Artificial neural network (ANN) analysis

FCCCD matrix, and experimental data (Table 1), were subjected to ANN analysis. The FCCCD data were divided into sections for training (to develop neural values and reduce prediction errors), validation (to select the optimal model and terminate training), and testing (for evaluation of ANN prediction potential). The ANN architecture is composed of an input layer with the five independent factors (five neurons), an output layer that has only one neuron (CNPs biosynthesis with *Olea* *europaea* leaves extract, mg/mL), and 20 hidden layers that were examined using several criteria, including the number of neurons, holdback propagation ratio, and learning rates. The trial-and-error procedure was used for model selection, and evaluation of machine learning efficacy, which was determined based on RMSE, MAD, SSE, and R^2^ tests. The fitness of FCCCD and ANN models was compared with the corresponding experimental values.

**Table 1:** FCCCD matrix mean actual and predicted values of chitosan nanoparticles biosynthesis using *Olea* *europaea* leaves extract.

Std	Run	Type	X_1_	X_2_	X_3_	X_4_	X_5_	Actual	Chitosan nanoparticles biosynthesis (mg/mL)			
									FCCCD		ANN	
									Predicted	Residuals	Predicted	Residuals
46	1	Center	0	0	0	0	0	14.92	15.48	-0.56	15.53	-0.61
5	2	Fact	−1	-1	1	−1	−1	10.91	10.42	0.49	10.88	0.03
1	3	Fact	−1	−1	−1	−1	−1	8.99	9.94	−0.95	9.14	−0.14
10	4	Fact	1	−1	−1	1	−1	11.09	11.16	−0.07	11.15	−0.06
24	5	Fact	1	1	1	−1	1	17.32	16.86	0.45	17.31	0.01
42	6	Axial	0	0	0	0	1	15.12	14.45	0.67	15.09	0.03
21	7	Fact	−1	−1	1	−1	1	9.72	9.28	0.44	9.58	0.14
31	8	Fact	−1	1	1	1	1	16.52	16.86	−0.34	16.73	−0.21
48	9	Center	0	0	0	0	0	15.09	15.48	−0.39	15.53	−0.44
36	10	Axial	0	1	0	0	0	18.28	17.92	0.36	18.36	−0.08
18	11	Fact	1	−1	−1	−1	1	10.53	9.81	0.72	10.42	0.11
6	12	Fact	1	−1	1	−1	−1	10.84	10.74	0.10	10.87	−0.03
11	13	Fact	−1	1	−1	1	−1	18.20	18.59	−0.39	18.20	0.00
37	14	Axial	0	0	−1	0	0	15.22	15.65	−0.42	15.20	0.02
3	15	Fact	−1	1	−1	−1	−1	18.76	17.9	0.86	18.78	−0.02
22	16	Fact	1	−1	1	−1	1	8.94	9.97	−1.03	9.13	−0.19
7	17	Fact	−1	1	1	−1	−1	17.04	17.82	−0.78	17.06	−0.02
13	18	Fact	−1	−1	1	1	−1	11.35	10.69	0.66	11.10	0.25
45	19	Center	0	0	0	0	0	15.44	15.48	−0.04	15.53	−0.09
43	20	Center	0	0	0	0	0	16.05	15.48	0.57	15.53	0.52
8	21	Fact	1	1	1	−1	−1	17.82	17.83	−0.01	18.10	−0.28
49	22	Center	0	0	0	0	0	15.16	15.48	−0.32	15.53	−0.37
29	23	Fact	−1	−1	1	1	1	8.66	9.5	−0.84	8.91	−0.25
50	24	Center	0	0	0	0	0	16.46	15.48	0.98	15.53	0.93
33	25	Axial	−1	0	0	0	0	15.48	15.73	−0.25	15.28	0.20
15	26	Fact	−1	1	1	1	−1	18.59	18.26	0.33	18.66	−0.07
19	27	Fact	−1	1	−1	−1	1	16.46	16.53	−0.08	16.49	−0.03
12	28	Fact	1	1	−1	1	−1	19.41	18.97	0.43	19.31	0.10
40	29	Axial	0	0	0	1	0	14.92	15.29	−0.36	14.94	−0.02
41	30	Axial	0	0	0	0	−1	14.87	15.54	−0.67	14.96	−0.09
14	31	Fact	1	−1	1	1	−1	10.89	11.05	−0.15	10.86	0.03
26	32	Fact	1	−1	−1	1	1	10.30	10.32	−0.02	10.25	0.05
47	33	Center	0	0	0	0	0	14.83	15.48	−0.65	15.53	−0.70
20	34	Fact	1	1	−1	−1	1	16.28	17.25	−0.97	16.36	−0.08
30	35	Fact	1	−1	1	1	1	10.53	10.23	0.30	10.36	0.17
39	36	Axial	0	0	0	−1	0	15.17	14.81	0.36	15.13	0.04
34	37	Axial	1	0	0	0	0	16.51	16.27	0.24	16.35	0.16
23	38	Fact	−1	1	1	−1	1	16.42	16.48	−0.06	16.34	0.08
25	39	Fact	−1	−1	−1	1	1	9.52	9.25	0.26	9.49	0.03
16	40	Fact	1	1	1	1	−1	17.78	18.3	−0.52	17.73	0.05
4	41	Fact	1	1	−1	−1	−1	18.46	18.24	0.22	18.59	−0.13
32	42	Fact	1	1	1	1	1	17.81	17.28	0.53	17.74	0.07
38	43	Axial	0	0	1	0	0	15.98	15.56	0.42	15.84	0.14
44	44	Center	0	0	0	0	0	15.91	15.48	0.43	15.53	0.38
2	45	Fact	1	−1	−1	−1	−1	10.82	10.6	0.22	10.96	−0.14
27	46	Fact	−1	1	−1	1	1	17.56	17.17	0.39	17.49	0.07
28	47	Fact	1	1	−1	1	1	17.50	17.93	−0.43	17.45	0.05
9	48	Fact	−1	−1	−1	1	−1	10.70	10.47	0.23	10.75	−0.05
17	49	Fact	−1	−1	−1	−1	1	8.79	8.78	0.01	8.76	0.03
35	50	Axial	0	−1	0	0	0	10.05	10.41	−0.37	10.28	−0.23

Variable	Variable code	−1	0	1								
Chitosan conc. (%)	X_1_	0.5	1	1.5								
Leaves extract conc. (%)	X_2_	50	75	100								
Initial pH level	X_3_	4	4.5	5								
Temperature (°C)	X_4_	40	50	60								
Incubation time (min)	X_5_	60	90	120								

### Statistical analysis

The software Design Expert version 12 for Windows (https://www.statease.com/software/design-expert/), was used for of conducting the statistical analysis and designing the experiments. For the purpose of plotting the three-dimensional surface plots, the STATISTICA software (Version 8.0, StatSoft Inc., Tulsa, USA) (https://www.statsoft.de/de/software/statistica) was used. The artificial neural network (ANN) analysis was performed using JMP pro 14 Software (https://www.jmp.com/en_in/home.html).

### In vitro effect of CNPs on biofilm formation

By employing microdilution method, the inhibitory effect of chitosan-NPs with different concentrations (10, 20, 50, 100, 200, 500, 1000, 1500 μg/mL) was assessed. The microbial cultures of *P.* *aeruginosa* (ATCC 27853), *S.* *aureuse* (ATCC 25923) and *C.* *albicans* (ATCC 10231) as models for MDR Gram-negative, Gram-positive bacteria and yeast forming biofilm, respectively, were prepared by diluting a loopful of overnight culture in sterile Trypticase Soy Broth (TSB) supplemented with 1% w/v glucose (TSBG). 100 μL (1 × 10^6^ CFU/mL) of culture suspensions were dispensed in each well of 96-well-U-bottomed microtiter plate. Positive control wells contained the microbial cultures without treatment; and negative control wells contained uninoculated TSBG. Additionally, 10, 20, 50, 100, 200, 500, 1000, 1500 μg/mL of standard antibiotics (tetracycline and streptomycin for prokaryotes and nystatin for eukaryotes) were also included for comparison. The plate was incubated at 37 °C for 24 h under static conditions to permit the formation and maturation of biofilm. The microbial biofilms were quantified spectrophotometrically at 595 nm using a microtiter ELISA reader (Tecan Infinite M200, Switzerland), after number of processing steps including washing, staining with Hucker's crystal violet and fixation as described by Elyamny et al. ^40^. The percent of biofilm inhibition was determined relative to the amount of positive control biofilm as described in the following equation^40^:

2 $${\text{Biofilm\, inhibition }}\% \, = \,\left( {{\text{A}} - {\text{A}}_{{\text{o}}} } \right)/{\text{A}} \times 100$$

Where A and A_0_ pointed out to the absorbance of the positive control and the treated wells, respectively.

### Effect of chitosan-NPs on biofilm metabolic activity

The viability of biofilm cells and their respiratory activity were determined by using MTT colorimetric assay. Biofilm was formed as described above. Following overnight incubation, the contents of each well were decanted, and wells were rinsed three times with PBS to remove loosely adhered cells. About 200 μL of 0.25 mg/mL MTT solution (3-[4, 5-dimethylthiazol-2-yl]-2, 5-diphenyltetrazolium bromide was mixed with content of each well. The microtiter plate was gently shaken, covered (in darkness) then incubated at 37 °C for 2–3 h; following incubation, the solution was removed and 2% DMSO was added to dissolve insoluble purple formazan that formed as a result of the enzymatic hydrolysis of MTT by biofilm cells. The absorbance was measured at 570 nm by microtiter ELISA reader. The data were interpreted in terms of inhibition percentage following the previous equation (Eq. 2)^41^.

### Effect of chitosan-NPs on biofilm hydrophobicity

The influence of different concentrations of chitosan-NPs on biofilm hydrophobicity was determined via microbial adherence to hydrocarbons (MATH) assay. The biofilms of examined strains were grown overnight and washed following the exact procedure described by Elyamny et al. ^40^. About 200 μL of PBS was added to the adhered biofilms and subjected to dislodging from the wells by vigorous scraping using a pipette tip; then, the scrapped biofilm in 3 mL PBS buffer were vortexed vigorously for 3 min to disrupt the biofilms. Thereafter, 0.4 mL of xylene (hydrocarbon) was mixed vigorously by vortex with 2 mL of biofilm suspensions for 2 min and incubated for 15 min at room temperature to allow the separation of aqueous phase and hydrocarbon of the mixtures. The optical density (OD) of the aqueous phase was measured at 600 nm to assess the hydrophobicity index (HI) or cell adhesion to hydrocarbons percentage as follows:

3 $${\text{Hydrophobicity \, index }}\left( {{\text{HI}}} \right)\, = \,\left( {{\text{OD6}}00{\text{ nm positive control}}} \right){-}\left( {{\text{OD6}}00{\text{ nm treated}}} \right)]/ \, \left( {{\text{OD6}}00{\text{ nm positive control}}} \right)\, \times \,{1}00.$$

All experiments were repeated trice to verify the results. The degree of hydrophobicity was classified as hydrophilic, moderately hydrophobic or strongly hydrophobic within percentage adhesion values of < 20%, 20–50% and > 50%, respectively^42^.

### Effect of chitosan-NPs on biochemical composition of the biofilm

The biofilm matrix of the examined strains was isolated from the microtiter plate as formerly mentioned in hydrophobicity experiment. The collected supernatants containing the adherent biofilm were subjected to quantification of total protein and total carbohydrate. The protein content was quantified using Bradford method and BSA as a standard; the carbohydrate content was determined using phenol–sulfuric assay and glucose as a standard, following protocols described in details by Shawki et al.^41^.

### Data analysis

All assays were carried out in triplicate, the data were expressed as means ± SEM (Standard Error of Mean) and their statistical analyses were performed via Minitab-14 software (Minitab Inc., Pennsylvania, USA) through one-way ANOVA with Tukey’s post hoc. Statistical significance was accounted at the *P* value recording less than 0.05.

**Figure 1:**
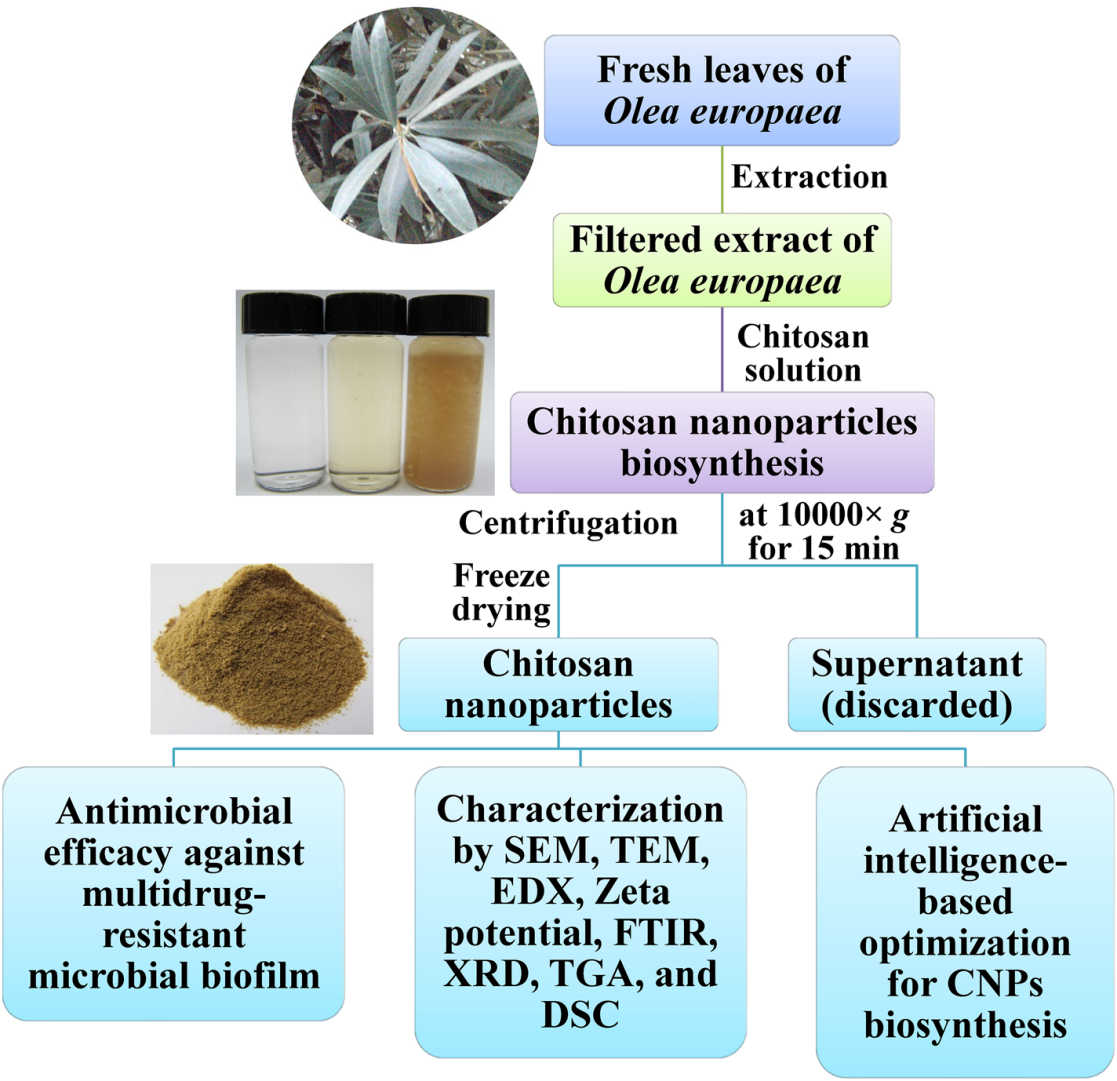
Schematic representation of the research process.

Figure 1 presents a schematic representation of the research process in this study.

## Results and discussion

**Figure 2:**
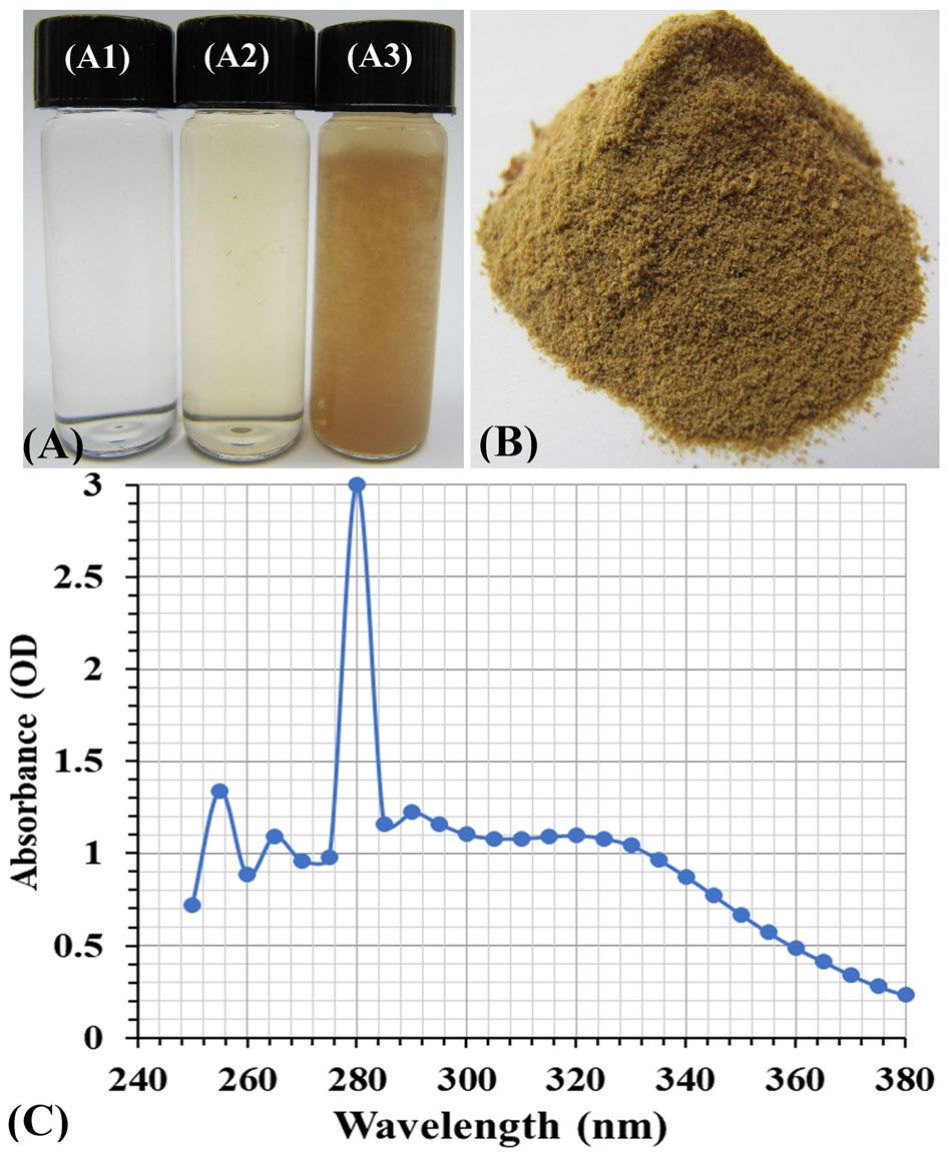
(**A**) Vials of chitosan solution (A1), *Olea* *europaea* leaves extract (A2), and the biosynthesized CNPs (A3), (**B**) lyophilized chitosan nanoparticles and, (**C**) UV/visible spectrum of the biosynthesized CNPs.

The production of chitosan nanoparticles has been accomplished by the application of a variety of methodologies. In addition to the particle size, the stability and safety of the CNPs are important aspects that should be taken into consideration when selecting a suitable preparation method. The green biosynthesis of CNPs offers many advantages, including being a one-step procedure, environmentally benign, non-toxic, and requiring less energy. Additionally, the biosynthesized CNPs are more stable^6^. The green biosynthesis of CNPs was accomplished with the help of microorganisms such as actinomycetes^6^, and fungi^43^. In addition, secondary metabolites present in aqueous extracts of plant leaves were applied as a reducing agent in the biosynthesis of nanoparticles^32,44^. Khandel and Shahi^45^; Chandran et al.^33^ and stated that the biological molecules like enzymes and proteins can act as either stabilizers or reducers, or perhaps both of these roles during the biosynthesis process of nanoparticles. In this study, an eco-friendly, cost effective, biosafe nanoparticles biosynthesis protocol using *Olea* *europaea* leaves extract was used. *Olea* *europaea* leaves extract contains high amount of phenolics and flavonoids which are considered antioxidant agents^46^. Antioxidants may have an effect as reducing agents in the sample that result in reduction of the Fe^3+^/ferricyanide complex to the ferrous form^46^. Similarly, *Olea* *europaea* leaves extract can act as a reducing agent for transition of chitosan molecules to their corresponding chitosan nanoparticles. Figure 2A shows leaves’ extract, chitosan solution, and the biosynthesized CNPs. Figure 2B shows the lyophilized biosynthesized CNPs using *Olea* *europaea* leaves extract.

## Characterization of CNPs biosynthesized using *Olea europaea* leaves extract

### UV/visible spectrum of CNPs

Optizen Pop-UV/Vis spectrophotometer was used to scan the biologically synthesized CNPs over 240 to 380 nm to determine the absorbance peak. Figure 2C depicts the scan spectrum of CNPs, which reveals a distinct strong absorption peak at 280 nm with an absorbance value of 3, attributed to the number of nanoparticles produced in response to reduction power of *Olea* *europaea* leaves extract. These findings are in agreement with those that were found by Duraisamy et al.^47^ who reported that the UV–visible spectrum of chitosan nanoparticles ranged between 200 and 322 nm due to the presence of the CO groups. The present findings are also in agreement with those of Sathiyabama and Parthasarathy^48^, who used proteins derived from *Penicillium* *oxalicum* to produce chitosan nanoparticles with a sharp peak at 285 nm.

### Electron microscopy examination

**Figure 3:**
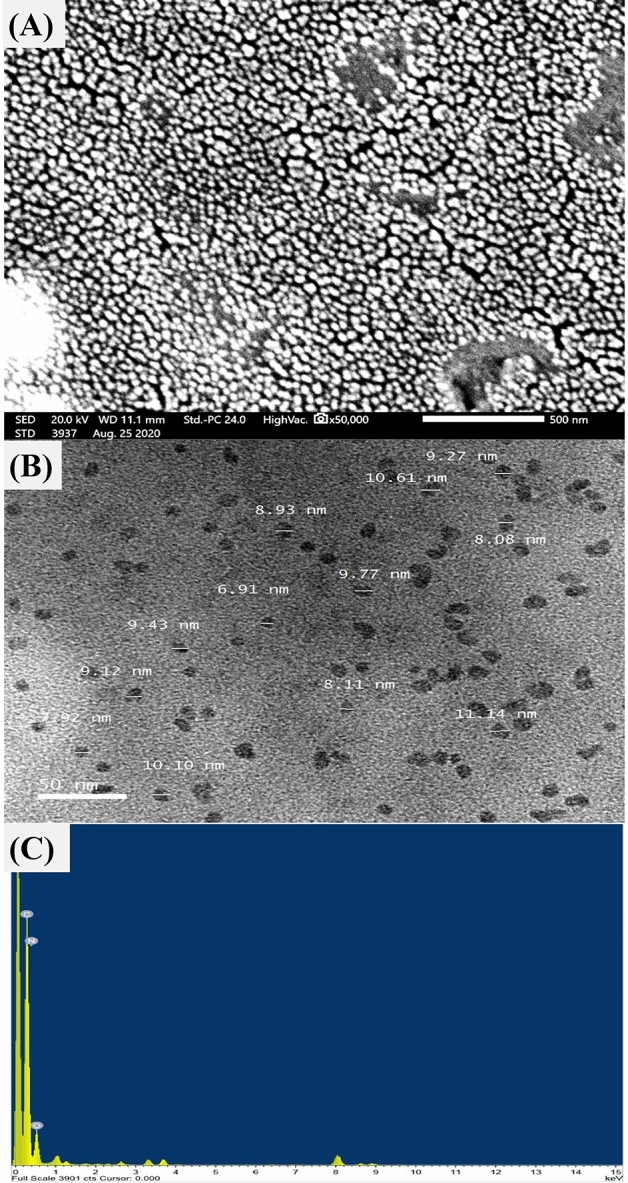
The biosynthesized CNPs using *Olea* *europaea* leaves extract, as detected by the micrographs of SEM (**A**), TEM (**B**) and EDX (**C**).

For many years, both scanning electron microscope (SEM) and transmission electron microscope (TEM) have been applied for the examination of nanoparticle size, shape, aggregation, dispersion and composition of the studied material. Transmission and scanning electron microscopy are effective methods for determining nanostructure morphology and size^49^. The SEM analysis of CNPs revealed homogenous spherical-like particles, as depicted in Fig. 3A. TEM analysis is an excellent tool for providing imaging, elemental and chemical characterization of nanoparticles^50^, including imaging, diffraction, and microanalytical data, to provide extensive insights into the characteristics and behavior of nanostructured materials. Figure 3B shows the size of CNPs ranging from 6.91 to 11.14 nm, with a relatively coarse texture and no evidence of aggregation. In pharmaceutical applications, the particle size of chitosan nanoparticles has a great influence on their characteristics. A smaller particle size can encapsulate a greater quantity of therapeutic substances, enhance the drug's stability and bioavailability, and enable prolonged administration^51^. The smaller particle size of CNPs also enhances drug delivery and thus efficacy, since it is easier to transport through biological membranes^51^.

Energy-dispersive X-ray spectroscopy (EDX) was applied to study the purity and to determine the structure of the sample^6^. Figure 3C shows EDX spectrum of the biosynthesized chitosan nanoparticles sample. EDX analysis showed a very homogenous elemental component of native chitosan (carbon, nitrogen, and oxygen). In the EDX spectrum the peak at 0 keV is the noise peak or zero energy strobe peak.

**Figure 4:**
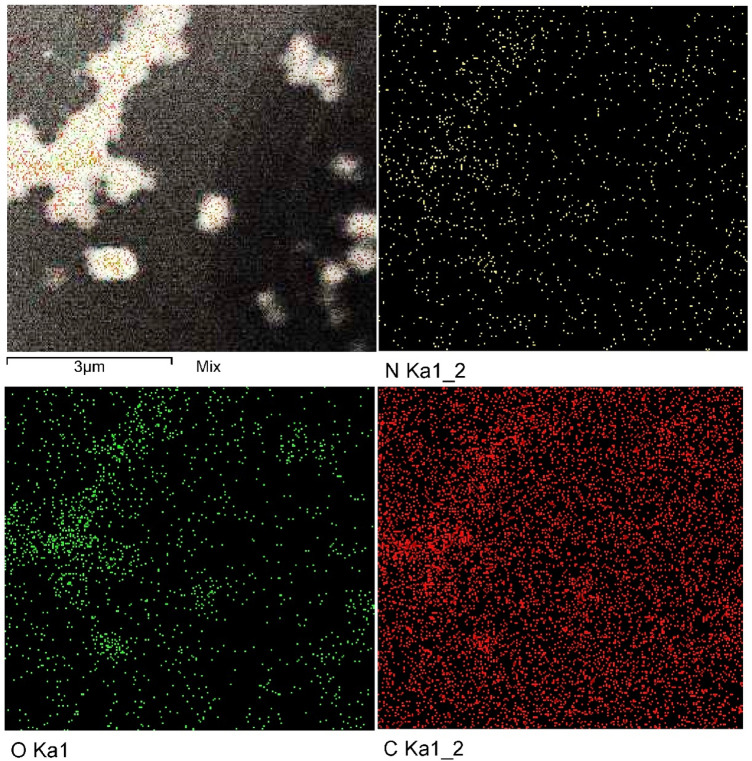
Mapping analysis of the biosynthesized CNPs using *Olea* *europaea* leaves extract.

Mapping analysis of CNPs was carried out to investigate the pattern of biosynthesized CNPs distribution. TEM elemental mapping analysis results illustrate the whole distribution of CNPs and its components (carbon, nitrogen and oxygen) (Fig. 4). The individual O, C, and N components of CNPs are dispersed and distributed in an identical manner.

### Zeta (ζ) potential analysis

**Figure 5:**
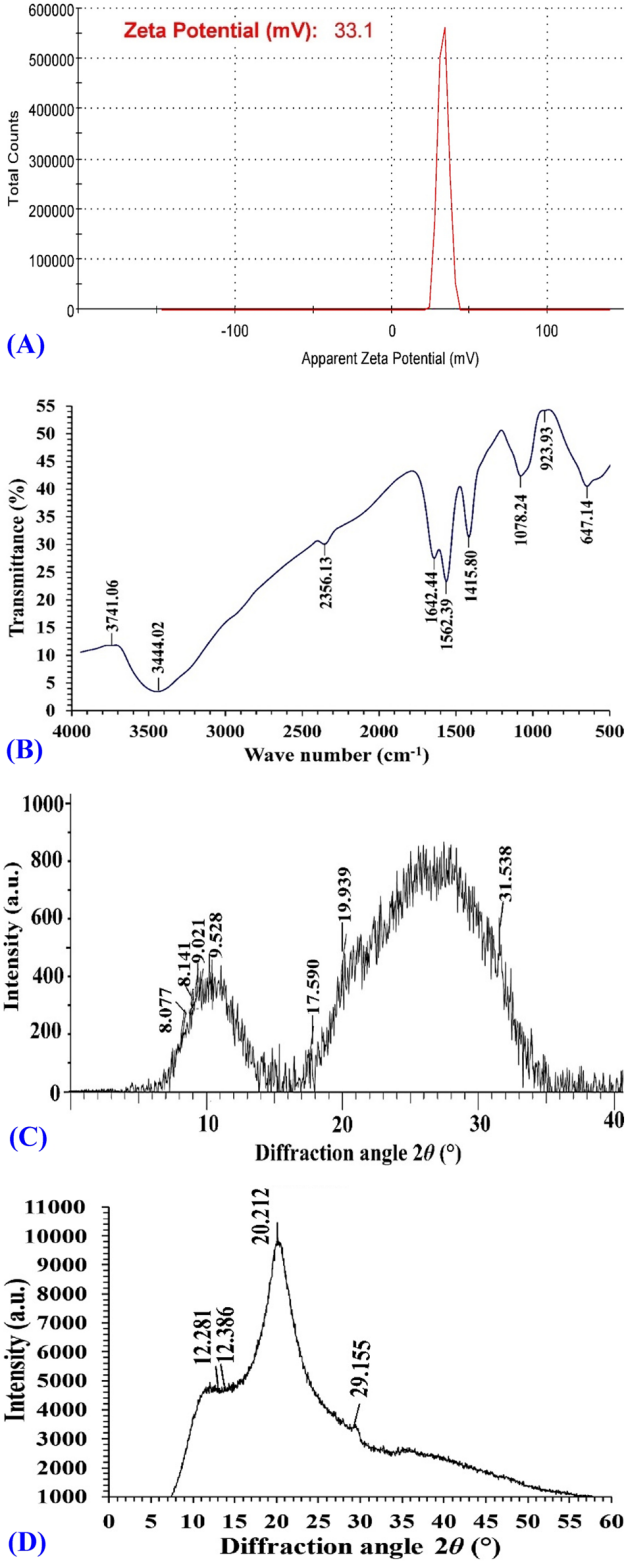
Analyses of the biosynthesized CNPs with (**A**) ζ-potential, FTIR (**B**), XRD (**C**).

The zeta potential of a particle is a parameter that represents particle charge and indicates the particle's stability. If each particle in suspension has a large zeta potential that is either negative or positive, they will tend to repel each other and will not aggregate. In contrast, there is no force preventing particles with a low zeta potential from aggregating and flocculating. Zeta potential value is crucial for understanding and predicting particle interactions in suspension^52^. The ζ-potential (Fig. 5A) shows a sole peak, representing homogenousity and stability of biosynthesized CNPs, which had a positive charge with ζ-potential of + 33.1 mV. This is in agreement with Kheiri et al.^53^, which reported that zeta potentials of the nanoparticles formed were positive due to residual protonated amine groups, and Yien et al.^54^ reporting that zeta potential of chitosan nanoparticles ranged from + 22 to + 55 mV and that their inhibitory effect was influenced by particle size and zeta potential. Similarly, Qi et al.^55^ found that the surfaces of chitosan nanoparticles had a positive charge of about 51 mV. The surface zeta potential of CNPs was determined to be approximately + 31 mV^56^.

### Fourier transform infrared (FTIR) analysis

FTIR analysis was conducted for characterization and identification of the functional groups found in the biosynthesized chitosan nanoparticles using *Olea* *europaea* leaves extract (Fig. 5B). The presence of capping groups on the surface of CNPs, which serve to stabilize them and prevent aggregation, was confirmed by FTIR analysis. The spectral band 3741 cm^−1^ is attributed to the free- and hydrogen-bonded-OH stretches as reported by Jiang et al.^57^. The peak at 2356 cm^−1^ is assigned to CO_2_^58^. The peaks at 1642 and 1562 cm^−1^ are attributed to C=O stretching of the amide group (CONH_2_) and groups of protonated amine (NH_2_), respectively. CNPs exhibited bending vibrations of the methyl group (C-H bending, alkane) at a wavelength of 1415 cm^−1^. The stretch vibration of CO groups (COH and COC) in the oxygen bridge, which is generated by chitosan deacetylation causes absorption at 1078 cm^−1^. In addition, spectral peaks in the range of 848–949 cm^−1^ are attributed to stretching vibration of C–O, which indicate the existence of mannuronic and uronic acids^59^. According to Flórez-Fernández et al.^60^, peaks located around 600 cm^−1^ could be the result of symmetric or asymmetric O=S=O deformation. The small peaks located at the end of the FTIR spectra correspond to the wagging of the saccharide structure of chitosan^32^.

### X-ray diffraction

The X-ray diffraction (XRD) examination is helpful in defining the crystalline structure of samples in terms of their physical properties. In this study, XRD was applied for examination of CNPs in terms of crystalline structure. In XRD, a material is first irradiated with incident X-rays, and then the intensities and scattering angles of the X-rays that escape the sample are measured^61^. The XRD pattern of CNPs sample showed seven distinctive peaks at 2*θ* which were at 8.077, 8.14, 9.02, 9.52, 17.59, 19.93 and 31.53° (Fig. 5C) indicating a shift from the normal chitosan peaks (Fig. 5D) which showed four distinctive peaks at 2*θ* at 12.281, 12.386, 20.212 and 29.155°. The XRD spectra of CNPs does not show the normal chitosan peaks due to the effect of *Olea* *europaea* leaves extract, indicating the formation of the NPs. Lazaridou et al.^62^ reported that the neat CS exhibited the weak diffraction peaks centered at diffraction angle 2*θ* = 11.9° and sharp diffraction peaks at 2*θ* = 20° were indicative of the high degree of crystallinity morphology of chitosan. Rasaee et al.^63^ reported that the CNPs exhibited diffraction peaks at 2θ = 10° (weak diffraction peak) and 20° (strong diffraction peak), revealing that the chitosan had a high degree of crystallinity. The crystalline structure of chitosan nanoparticles was demonstrated by XRD patterns that displayed strong peak at angle of about 20° (19.93) (Fig. 5C). Similar results were obtained by Divya et al.^25^, who stated that the chitosan diffraction pattern exhibits peaks at 2*θ* = 9.28° and 20.18°, reflecting the crystalline structure II. In the XRD diffraction patterns of the chitosan and CNPs, the peaks at 2*θ* of 10.18° and 20.26° were detected, reflecting the (020) hydrated crystalline structure and (110) anhydrous crystalline structure, respectively^64^. This implies the presence of a crystalline phase in the CNPs produced. On the other hand, XRD of CNPs display two peaks at 2θ = 17° and 25°, which indicated a shift from the peaks of normal chitosan. The XRD patterns of chitosan showed three strongest distinctive peaks located at 2θ = 20.4°, 26.4° and 29.5°^65^.

### CNPs' thermal characteristics

**Figure 6:**
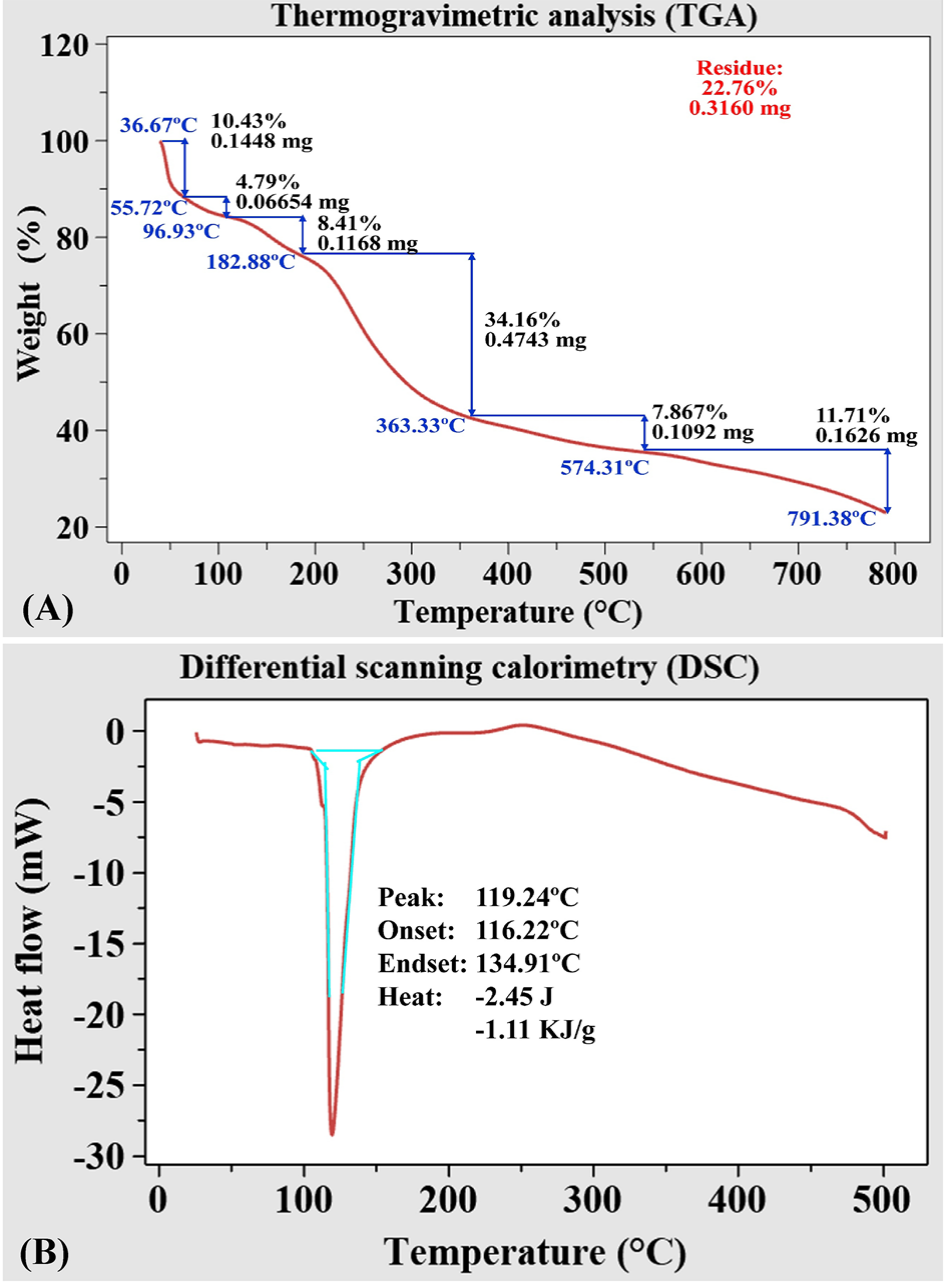
Thermograms of TGA (**A**), DSC (**B**) of the biosynthesized CNPs using *Olea* *europaea* leaves extract.

The thermal characteristics of biosynthesized CNPs using *Olea* *europaea* leaves extract were obtained using two main ways: TGA (thermogravimetric analysis) and DSC (differential scanning calorimetry). Thermogravimetric analysis is used for CNPs characterization and investigation of thermal properties (between room temperature and 800 °C). Figure 6A showed that rapid initial mass reduction (-10.43%) can be easily detected when the temperature rises from 36.67 to 55.72 °C, leaving 0.1448 mg of the sample as residue due to breakdown of volatile units as reported by Vijayalakshmi et al.^66^ or dehydration of the saccharide rings process which does not result in chemical reactions or lead to any structural alterations^67^. Mass reduction (- 4.79%) was observed when the temperature was raised from 55.72 to 96.93 °C, leaving 0.06654 mg of the sample as residue. At temperatures below 100 °C, the chitosan sample exhibited a loss of mass due to evaporation of water^68^. At higher temperatures, weight loss of biologically synthesized CNPs was observed with multistage decomposition. The temperature range of 182.88 to 363.33 °C resulted in the greatest weight loss (34.16 percent (0.4743 mg) due to chitosan thermal degradation^67^, while the lowest weight loss (7.887%) was measured over a range of 363.33–574.31 °C. At 791.38 °C, approximately 77.372% of the CNPs sample get destroyed, leaving 22.628% of the sample with higher thermal stability remaining. Increased thermal stability suggests that crosslinking has caused the hydrogel network to become stronger and more rigid^66^. TGA alone may not be sufficient to identify destroyed products, therefore, DSC is essential as well as TGA to ascertain the presence of intermediate destructed products^69^.

Differential scanning calorimetry (DSC) is a technique for measuring the thermal effects of phase transitions and chemical processes as a function of temperature. The DSC analysis was performed at multiple rates of heating to show the amount of variation in the CNPs heat flow as a result of temperature (Fig. 6B). With the change in the thermodynamic system, one endothermic peak at 119.24 °C was identified through the range of 116.22–134.91 °C, requiring −2.45 J/g CNPs is in accordance with Zhao et al*.* ^70^ who reported that the DSC curve of the blank chitosan nanoparticle showed an endothermic peak near 90 °C.

### Statistical optimization of chitosan nanoparticles biosynthesis using *Olea europaea* leaves extract using face centered central composite design (FCCCD)

The size, distribution, and quantity of synthesized chitosan nanoparticles are influenced by many variables such as incubation time, temperature, pH and reactant concentration^71^. In the current study, the effects of five factors on CNPs biosynthesis (as a response) were explored. These five variables were the concentration of chitosan, the concentration of leaves extract, the initial pH level, temperature, and the length of incubation time. Using the FCCCD, the bioprocess variables of CNPs biosynthesis were optimized to maximize CNPs biosynthesis and to investigate the individual, interaction, and quadratic effects of process variables on CNPs biosynthesis using *Olea* *europaea* leaves extract. FCCCD of fifty trials was used in order to determine the optimal values for the variables of interest. Table 1 presents the FCCCD which had ten axial points, eight center points, and thirty-two factorial points. To determine the experimental errors, eight duplicates at the center locations were carried out. Table 1 illustrates the design matrix containing the five investigated variables, their actual and coded levels, as well as the experimental and predicted biosynthesized CNPs values (mg/mL) and their residual values.

The obtained results of FCCCD experiments for the biosynthesized CNPs using *Olea* *europaea* leaves extract show considerable variations in the biosynthesized CNPs based on the five variables. According to the measured data, the biosynthesized CNPs concentrations ranged between 8.66 and 19.41 mg/mL. The highest level of the biosynthesized CNPs (19.41 mg/mL) was recorded in the run 28 in which concentration of chitosan was 1.5%, concentration of leaves extract was 100%, pH was 4, temperature was 60 °C, and the incubation time was 60 min. Whereas, the lowest biosynthesized CNPs (8.66 mg/mL) achieved in the run number 23 in which concentration of chitosan was 0.5%, concentration of leaves extract was 50%, pH was 5, temperature was 60 °C, and the incubation time was 120 min.

### Multiple regression analysis and analysis of variance (ANOVA) 

FCCCD data for the biosynthesized CNPs using *Olea* *europaea* leaves extract was statistically analyzed by multiple regression statistical analysis and ANOVA. The calculations listed in Table 2 including the coefficient estimate values, R^2^ value, predicted R^2^ value, adj R^2^ value, *P*-value (probability value) lack of fit and *F*-value (Fisher value) have been assessed for the model reliability. In addition, the linear, interaction, and quadratic effects of the five selected process factors were evaluated^72^.

**Table 2:** Analysis of variance for chitosan nanoparticles biosynthesis using *Olea* *europaea* leaves extract obtained by FCCCD.

Source of variance		Sum of square	Degrees of freedom	Mean of square	*F*-value	*P*-value	Coefficient estimate
Model		522.240	20	26.110	59.580	< 0.0001	15.48
Linear effect	X_1_	2.460	1	2.460	5.620	0.0246	0.27
	X_2_	478.740	1	478.740	1092.280	< 0.0001	3.75
	X_3_	0.061	1	0.061	0.140	0.7113	−0.04
	X_4_	1.910	1	1.910	4.360	0.0457	0.24
	X_5_	10.140	1	10.140	23.130	< 0.0001	−0.55
Interaction effect	X_1_X_2_	0.193	1	0.193	0.440	0.5126	−0.08
	X_1_X_3_	0.222	1	0.222	0.506	0.4824	−0.08
	X_1_X_4_	0.003	1	0.003	0.006	0.9369	0.01
	X_1_X_5_	0.277	1	0.277	0.632	0.4331	0.09
	X_2_X_3_	0.614	1	0.614	1.400	0.2461	−0.14
	X_2_X_4_	0.056	1	0.056	0.128	0.7233	0.04
	X_2_X_5_	0.080	1	0.080	0.182	0.6726	−0.05
	X_3_X_4_	0.134	1	0.134	0.305	0.5852	−0.06
	X_3_X_5_	0.001	1	0.001	0.003	0.9588	0.01
	X_4_X_5_	0.006	1	0.006	0.013	0.9096	−0.01
Quadratic effect	X_1_^2^	0.663	1	0.663	1.510	0.2287	0.52
	X_2_^2^	4.280	1	4.280	9.760	0.004	−1.32
	X_3_^2^	0.036	1	0.036	0.083	0.7755	0.12
	X_4_^2^	0.460	1	0.460	1.050	0.314	−0.43
	X_5_^2^	0.578	1	0.578	1.320	0.2603	−0.48
Error effect	Lack of fit	10.250	22	0.466	1.330	0.3686	15.48
	Pure error	2.460	7	0.351			0.27
R^2^	0.9762	Std. dev	0.662				
Adj R^2^	0.9599	Mean	14.4				
Pred R^2^	0.9217	C.V. %	4.6				
Adeq precision	23.76	PRESS	41.91				

*F* Fishers's function, *P* level of significance, *C.V*. coefficient of variation.

*Significant values.

The current regression model has R^2^ value = 0.9762. When the coefficient of determination (R^2^) is higher than 0.9, the model was considered to be strongly correlated^73^. Our present study implies that R^2^ value of the model used for the biosynthesized CNPs is 0.9762, reflecting that 97.26% of variance in the biosynthesized CNPs was attributed to the independent factors, the model is not capable of describing only 2.74% of the total variance.

The value of Adj R^2^ (0.9599) is very high, confirming the accuracy of the model (Table 2). The value of predicted R^2^ = 0.9217 was in a reasonable agreement with adj. R^2^ value which proved a high compatibility among observed and predicted values of the response^74^.

The adequate precision value of the present model was 23.76. The model's mean and standard deviation are 14.4 and 0.662, respectively (Table 2). Adequate precision indicates the noise level, level > 4 (23.76) reveals high model accuracy, suggesting appropriate design space for optimizing CNPs biosynthesis at the various levels of the evaluated parameters^75^. Statistically analyzed data of CNPs biosynthesis reveals that coefficient of variation percent (C.V.) = 4.6% which is relatively low and reflects high level of accuracy, reliability and precision of the experimental trials^76^. Data also demonstrates lower standard deviation (0.662).

Furthermore, the coefficient estimate revealed positive or negative effects on CNPs biosynthesis. A large estimated effect, whether positive or negative, indicates that the independent factors have a significant influence on the response. If the predicted effect of any tested variable has a positive sign, it shows that production increases at high levels of that variable. Conversely, when the sign is negative, it suggests that production is higher when the variable is low^77,78^. Antagonism (negative coefficient) and synergism (positive coefficient) are interactions between two variables. Positive coefficients for X_1_, X_2_, X_4_ indicated a linear effect of these variables in the increase of CNPs biosynthesis. Furthermore, negative coefficients for X_3_, X_5_ indicated a linear effect of these variables in the decrease of CNPs biosynthesis.

Probability values (*P*-values) and *F*-values (Table 2) were used to check the significance of each coefficient, which is needed to evaluate the significance of the variables and interpret their mutual interactions. The significance of the coefficient increased as the *P*-values declined. In addition, process variables with *P*-values less than or equal to 0.05 were deemed to have a significant influence on the response^79^. The model's *F*-value was 59.58, as well as the *P*-value was less than 0.0001, indicating that the model was highly significant. Based on the *P*-values of the coefficient, it can be concluded that the linear effects of chitosan concentration (X_1_), concentration of leaves extract (X_2_), temperature (X_4_) and incubation time (X_5_) and quadratic effect of X_2_ (concentration of leaves extract) are significant for chitosan nanoparticles biosynthesis using *Olea* *europaea* leaves extract. This means that they act as limiting factors, and small variations in their levels will alter the rate of CNP biosynthesis.

The fit summary results shown in Table 3 used to determine which of the linear, 2FI, and quadratic models was the most appropriate polynomial model for the CNPs biosynthesis using *Olea* *europaea* leaves extract. The quadratic model is the adequate model for CNPs biosynthesis where lack of fit (*P*-value = 0. 3686; *F*-value = 1.33) is non-significant with higher values of the adj. R^2^ (0.9599) and predicted R^2^ (0.9217). The given data demonstrates that the model's lack-of-fit error did not reach the significance level, as indicated by a higher *P*-value. Furthermore, the model's coefficient of variation, accuracy, and standard deviation all demonstrated adequate performance^80^.

**Table 3:** Fit summary of FCCCD for chitosan nanoparticles biosynthesis using *Olea* *europaea* leaves extract.

Source	Sequential p-value	Lack of fit p-value	Adjusted R^2^	Predicted R^2^	
Linear	< 0.0001*	0.0654	0.9133	0.9039	
2FI	0.999	0.0329	0.8921	0.8381	
Quadratic	< 0.0001*	0.3686	0.9599	0.9217	

Source	Sum of squares	*df*	Mean square	*F-*value	*P-*value prob > *F*
Lack of fit tests					
Linear	39.18	37	1.06	3.02	0.0654
2FI	37.6	27	1.39	3.97	0.0329*
Quadratic	10.25	22	0.4661	1.33	0.3686

Model summary statistics					
Source	Standard deviation	R-squared	Adjusted R-squared	Predicted R-squared	PRESS
Linear	0.97	0.9222	0.9133	0.9039	51.41
2FI	1.09	0.9251	0.8921	0.8381	86.6
Quadratic	0.66	0.9762	0.9599	0.9217	41.91

*df* degree of freedom, *PRESS* sum of squares of prediction error, *2FI* two factors interaction.

*Significant values.

The mathematical relations among the independent factors and the responses are demonstrated by the following equation:

4 $${\text{Chitosan nanoparticles biosynthesis value}}\, = \,{15}.{48}\, + \,0.{\text{27 X}}_{{1}} \, + \,{3}.{\text{75 X}}_{{2}} - 0.0{\text{4 X}}_{{3}} \, + \,0.{\text{24 X}}_{{4}} - 0.{\text{55 X}}_{{5}} - 0.0{\text{8 X}}_{{1}} {\text{X}}_{{2}} - 0.0{\text{8 X}}_{{1}} {\text{X}}_{{3}} \, + \,0.0{\text{1 X}}_{{1}} {\text{X}}_{{4}} \, + \,0.0{\text{9 X}}_{{1}} {\text{X}}_{{5}} - 0.{\text{146 X}}_{{2}} {\text{X}}_{{3}} \, + \,0.0{\text{4 X}}_{{2}} {\text{X}}_{{4}} - 0.0{\text{5 X}}_{{2}} {\text{X}}_{{5}} - 0.0{\text{6 X}}_{{3}} {\text{X}}_{{4}} \, + \,0.0{\text{1 X}}_{{3}} {\text{X}}_{{5}} - 0.0{\text{1 X}}_{{4}} {\text{X}}_{{5}} \, + \,0.{\text{52 X}}_{{1}}^{{2}} - {1}.{\text{32 X}}_{{2}}^{{2}} \, + \,0.{\text{12 X}}_{{3}}^{{2}} - 0.{\text{43 X}}_{{4}}^{{2}} - 0.{\text{48 X}}_{{5}}^{{2}} .$$

Where Y is the predicted value of CNPs biosynthesis, chitosan concentration (X_1_), concentration of leaves extract (X_2_), initial pH level (X_3_), temperature (X_4_) and incubation time (min) (X_5_).

### Effects of process variables on CNPs biosynthesis using *Olea europaea* leaves extract (three-dimensional surface plots) 

**Figure 7:**
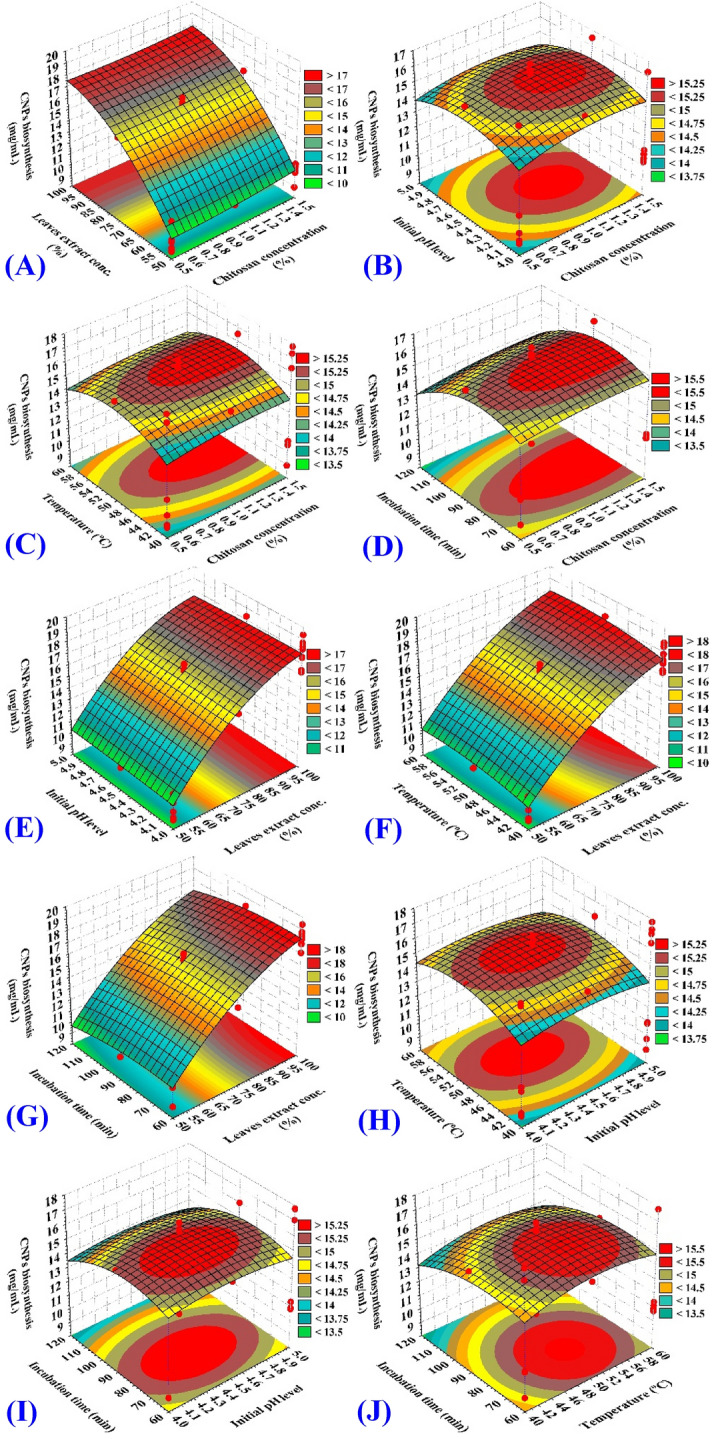
Three-dimensional surface plot of the biosynthesized CNPs using *Olea* *europaea* leaves extract, showing the interactive effects of the five tested variables.

Plots of the three-dimensional (3D) surface of the five independent factors were created in order to examine the dual effects of each pair of factors on CNPs biosynthesis using *Olea* *europaea* leaves extract (Fig. 7).

### Effect of chitosan concentration % on CNPs biosynthesis

Figure 7A–D depicts the three-dimensional response surface plots as function of the chitosan concentration % on the CNPs biosynthesis using *Olea* *europaea* leaves extract when interacting with the other variables: concentration of leaves extract (%) (X_2_), initial pH level (X_3_), temperature (°C) (X_4_) and incubation time (min) (X_5_); respectively. The plots reveal that the CNPs biosynthesis increased as chitosan concentration increased to the optimal level. Maximum CNPs biosynthesis (19.244 mg/mL) was obtained toward the high level of chitosan concentration (around 1.5%). Our findings are in agreement with those of Vaezifar et al.^81^, who found that a chitosan concentration at 1.5% was the best initial chitosan concentration to generate CNPs with the smallest size, as compared with greater concentrations. In contrast, Mahmoud et al.^82^ produced CNPs at a concentration of 2%., and Vaezifar et al.^81^, reported that optimal initial concentration of chitosan was 1.295 mg/mL. It is interesting to note that Sathiyabama and Parthasarathy^48^ reported that chitosan concentration of 0.5% was used for the synthesis of CNPs, and Kamat et al.^71^ stated that the highest production of nanoparticles could be obtained by using a concentration of chitosan of 0.8 mg/mL, the concentration of chitosan has significant impact on both the size and the yield of the nanoparticles^83^.

### Effect of concentration of leaves extract on CNPs biosynthesis

Figure 7A,E,F,G depicts the three-dimensional response surface plots as function of the concentration of leaves extract (%) (X_2_) on the CNPs biosynthesis when interacting with the other variable components: chitosan concentration (%) (X_1_), initial pH level (X_3_), temperature (°C) (X_4_) and incubation time (min) (X_5_); respectively. The plots reveal that the CNPs biosynthesis increased as the concentration of leaves extract (%) increased to the optimal level. Maximum CNPs yield (19.244 mg/mL) was obtained toward the high level of the concentration of leaves extract (%) (around 100%).

### Effect of initial pH level on CNPs biosynthesis

The 3D surface graphs (Fig. 7B,E,H,I) illustrated the effect of initial pH level on CNPs biosynthesis when interacting with the other variable components: chitosan concentration (%) (X_1_), the concentration of leaves extract (%) (X_2_), temperature (°C) (X_4_) and incubation time (min) (X_5_); respectively. The plots reveal that the CNPs biosynthesis increased by the rise of initial pH level. Maximum yield following CNPs biosynthesis was obtained toward the center point of initial pH level (around 4–4.5). Further increase or decrease led to the decrease in the CNPs biosynthesis. This is similar to the findings of Sathiyabama and Parthasarathy^48^, where the optimal pH for maximum CNPs biosynthesis was 4.8. Shu and Zhu^84^ also reported that the electrostatic interaction between the polyanions and chitosan during the formation of CNPs was dependent on the pH of the solution. The electrostatic interactions only occur in certain pH range which corresponds with the anion’s natural characteristics, for examples 1.0– 7.5 for CNPs-sulphate, 4.5–7.5 for CNPs-citrate and 1.9–7.5 for CNPs-TPP. Liu and Gao^85^ reported that pH also affects particle size and zeta potential of CNPs. The particle size growing rapidly from pH 1 to 3.5 and then decreased slowly from pH 3.5 to 5.5. The effect of pH on zeta potential was similar, with a slightly higher transition at pH 4, where zeta potential value increased between pH 1 and 4, then reduced slowly between pH 4 and 5.5. It was because in acidic condition the amine groups of chitosan were protonated which led to strong charge repulsion and molecule extension^86^.

### Effect of incubation period on CNPs biosynthesis

The 3D surface graphs (Fig. 7D,G,I,J) represent the three-dimensional response surface plots as function of the incubation period on the CNPs biosynthesis when interacting with the other three variable components: chitosan concentration (%) (X_1_), the concentration of leaves extract (%) (X_2_), initial pH level (X_3_) and temperature (°C) (X_4_), respectively. The plots reveal that the CNPs biosynthesis increased with the increase of the incubation period. Maximum yield following CNPs biosynthesis was obtained during 74.25 min of incubation period. Further increase or decrease led to the decrease in the CNPs yield. Our findings are consistent with those of El-Naggar et al. ^2^ who stated that the maximum CNPs biosynthetic yield, using *P.* *graveolens* leaves extract, was calculated to be 9.73 mg/mL after 57.53 min, while de Oliveira et al.^87^ reported that a 12-h incubation period was adequate for CNPs production. Sathiyabama and Parthasarathy^48^ synthesized CNPs at with a shorter time of 30 min using magnetic stirring. Saifful and Shahidan^88^ showed that increasing the incubation period to 18 h yielded nanoparticles with a larger average size as compared with the shorter incubation time (2 h).

### Effect of temperature on CNPs biosynthesis

The 3D surface graphs (Fig. 7C,F,H,J) depict the three-dimensional response surface plots as function of temperature on the CNPs biosynthesis when interacting with the other variable components: chitosan concentration (%) (X_1_), the concentration of leaves extract (%) (X_2_), initial pH level (X_3_) and incubation time (min) (X_5_), respectively. The plots reveal that CNPs yield increased as temperature increased to the optimal level. Maximum CNPs biosynthesis (19.244 mg/mL) was achieved at the temperature of approximately 54.27 °C. Temperature plays a crucial role in particle synthesis and shape/size regulation; it can greatly affect reaction rate and, consequently, particle properties^89^. Tsai et al.^90^ reported that the best temperature to produce the smallest CNPs by mechanical shearing were 45 °C (145 nm), followed by 4 °C (150 nm), and 25 °C (163 nm). In contrast, Kamat et al.^71^ stated that the highest yield of CNPs was produced at a temperature of 35 °C. Several studies reported that the CNPs formation also being affected by temperature^90^. Tsai et al.^90^ reported that the smallest CNPs when produced by the ionotropic gelation method at ambient temperature (25 °C) compared to higher temperature range (40–60 °C). CNPs obtained at higher temperature were easier to agglomerate as the result of the increased size and yield. On the other hand, Kamat et al.^71^ also reported that by using ionic gelation process for CNPs synthesis, lower temperature produced smaller particle size. CNPs produced at 4 °C were smaller (60–80 nm) and with narrower size distribution, compared to the ones produced at 35 °C which had larger size (130–190 nm) and broader size distribution, it was also easy to agglomerate.

### The model adequacy

**Figure 8:**
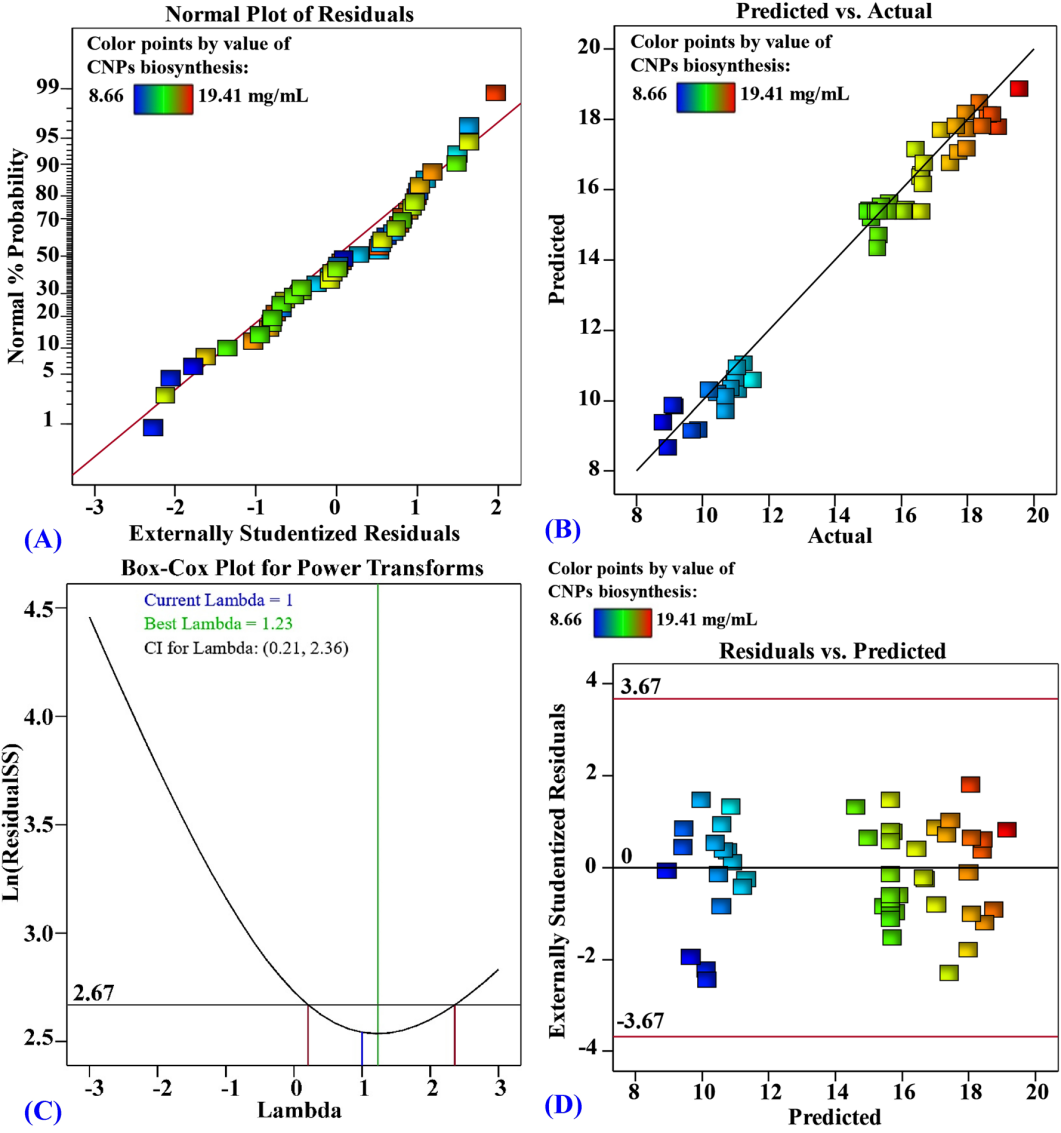
Normal probability plot of internally studentized residuals (**A**), plot of predicted versus actual (**B**), Box–Cox plot of model transformation (**C**), plot of internally studentized residuals versus predicted values (**D**) of the biosynthesized CNPs using *Olea* *europaea* leaves extract.

The normal probability plot of the residuals is a graphical technique used to assess whether or not a data set is normally distributed to certify the fitness of a model^91^. Residuals are variations between theoretical and experimental data, where small residuals indicate high model accuracy^92^. Figure 8A demonstrates that all points lie along the diagonal line, revealing that the regression model predictions agree with the actual results, confirming the accuracy of the model^93^. Figure 8B shows the actual values against predicted, for CNPs biosynthesis using *Olea* *europaea* leaves extract, demonstrating that all the experimental points are located very close to the prediction line, suggesting adequate fitting of the model to the experimental data^94^. Box-Cox plot of model transformation (Fig. 8C) is helpful for checking data which are not normally distributed. It shows that the Lambda (λ) optimal value of 1 is located between the two vertical red lines (the lowest and highest values of the 95% confidence values which are 0.21 and 2.36, respectively). This indicates that no data transformation was required, and that the model fits the obtained experimental results well^93^. Figure 8D represents the residuals against the predicted values for CNPs biosynthesis using *Olea* *europaea* leaves extract, showing that the residuals are located randomly around the zero-line, suggesting that the variance of the experimental results is constant for all values^95^. The current distribution pattern is sufficiently accurate to support the applicability of the FCCCD model.

### The artificial neural network (ANN) modelling prediction for CNP biosynthesis

The artificial intelligence-based approach was employed for analyzing, validating and predicting CNPs biosynthesis with *Olea* *europaea* leaves extract (Table 1). The construction or topology of artificial neural networks is regulated by two principal parameters including the number of layers and the number of nodes or neurons in each hidden layer. The network design of ANN modelling comprises learning and training processes, validation and verification of the final ANN model.

**Figure 9:**
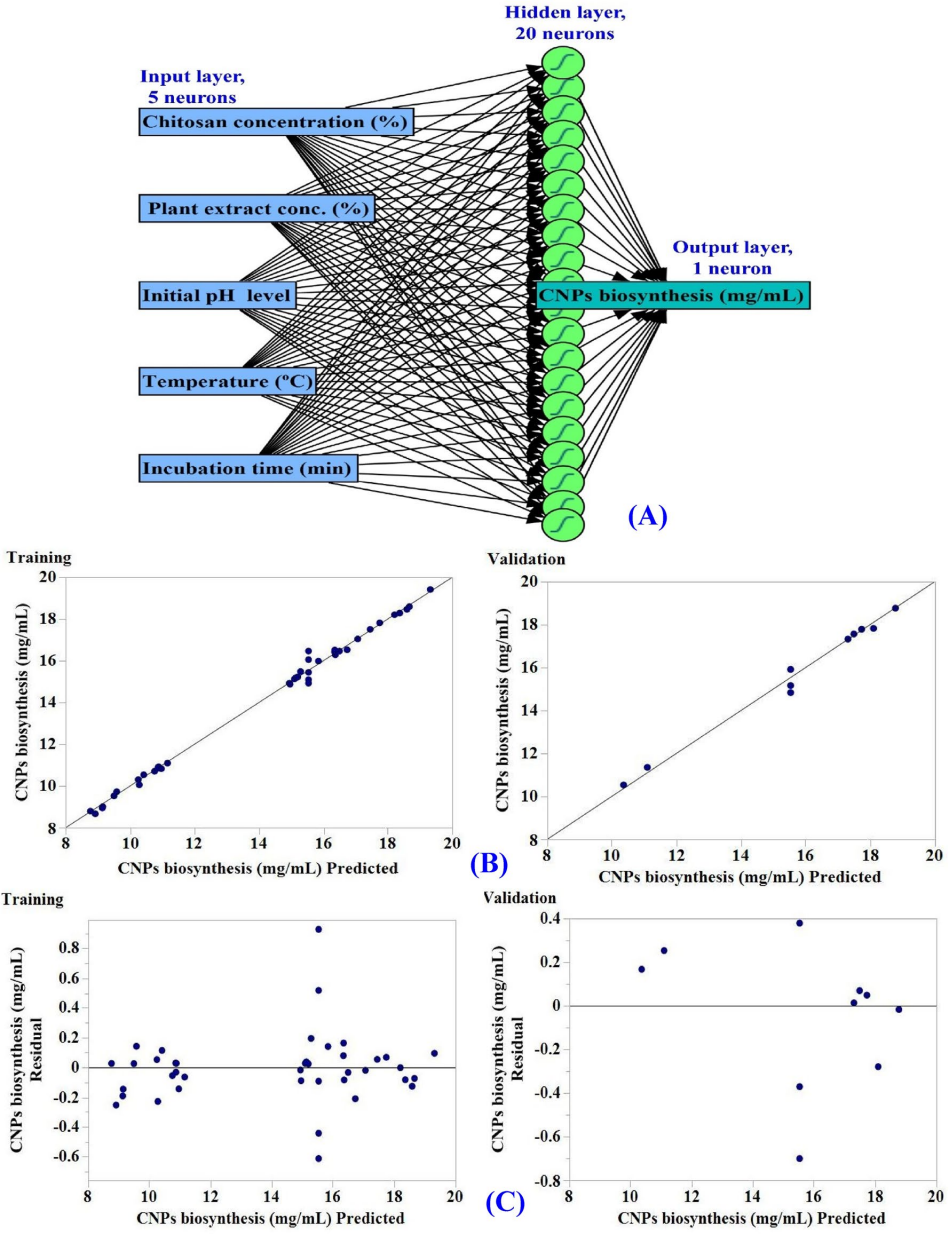
The final artificial neural network of the biosynthesized CNPs (**A**), the ANN predicted versus actual (**B**), the residuals versus ANN predicted (**C**) values of chitosan nanoparticles biosynthesis using *Olea* *europaea* extract.

ANN was used for optimizing of the process of chitosan nanoparticles biosynthesis using *Olea* *europaea* leaves extract. Simple neural network architecture has interconnected artificial neurons organized in three layers including input layer, hidden layers and output layer. In this study, one input layer accepts the initial data of the four independent factors (chitosan concentration, volume of plant extract, initial pH level, temperature, incubation time) for further processing in the system by the subsequent layers. Input nodes process the data, analyze or categorize it, and pass it to the subsequent layer. The hidden layer is set of 20-neurons between the input layer and the output layer. Hidden layer gets their data from the input layer, the data is changed and sent out to the output layer. One output layer that is the last layer of neurons to provides the final outcome of the artificial neural network's data processing results (CNPs biosynthesis, mg/mL) (Fig. 9A). The optimal ANN parameters were adjusted to number of tours (5000), model NTanH (20), a learning rate of 0.1 and a validation method (holdback, 0.2). The process of machine learning continued until the error values, such as mean absolute deviation (MAD), root mean square error (RMSE), and the sum of squared errors (SSE), reached their lowest values, in addition to the highest value of R^2^, for both training and validation processes (Table 4).

**Table 4:** ANN analysis and modeling comparison of predictive capability between FCCCD and ANN for CNPs biosynthesis using *Olea* *europaea* leaves extract.

Measure	ANN		Overall model performance		
	Training	Validation	Statistics	FCCCD	ANN
R^2^	0.9951	0.9866	R^2^	0.9762	0.9942
RMSE	0.2318	0.3084	RASE	0.5045	0.2488
MAD	0.1436	0.2298	AAE	0.4274	0.1604
SSE	2.1486	0.9510	Freq	50	50
Sum freq	40	10			

*RMSE* root mean squared error, *MAD* mean absolute deviation, *SSE* the sum of squares error, *RASE* root average squared error, *AAE* average absolute error for each model.

### Evaluation of ANN model

The predicted values of CNPs biosynthesis by ANN corresponding to each experimental result were given in Table 1. The predicted values of CNPs biosynthesis by ANN were drawn against the actual values (Fig. 9B). In both the training and validation phases, the points are gathered closer to the line showing the optimal prediction, indicating that the model is reliable. The scattering of residual data above and below the regression line revealed a normal scattering of the residuals (Fig. 9C) which support for the suitability of the ANN model.

### Comparison of prediction potential of ANN versus FCCCD

As illustrated in Table 1, the CNPs biosynthesis values predicted by ANN exhibit a more reasonable agreement with the experimental result, and the residuals values were lower than those obtained by the FCCCD model. The model’s performance was compared using the model comparison dialog in the JMP Pro14. For comparison, several error functions, as well as the R^2^ used to evaluate the prediction ability of the FCCCD and ANN. The most frequently employed functions for the comparison were R^2^, root average squared error (RASE) and average absolute error (AAE) for each regression model which are shown in Table 4. The prediction ability of the FCCCD and ANN was compared, the higher value of R^2^ (0.9942) along with lower value for RASE (0.2488) and lower value for AAE (0.1604) (Table 4) confirms ANN as the better model with a higher predictive capacity for the optimal levels of different physicochemical variables for CNP biosynthesis. It is likely to be the function of repeated training of the neurons for various physicochemical variables.

### Desirability function (DF)

The desirability function was performed in order to determine the best predicted conditions that would give maximum value of chitosan nanoparticle yield during biosynthesis. The key objectives of the experimental design and the DF were to identify the optimum predicted conditions and to maximize the responses^96^. Using the desirability function, the optimum conditions for maximum CNP biosynthesis with *Olea* *europaea* was determined theoretically and verified experimentally. The optimal theoretical conditions that maximize CNPs biosynthesis using *Olea* *europaea* leaves extract was determined to be: chitosan concentration (1%), concentration of leaves extract (100%), initial pH level 4.47, temperature 53.83°C and incubation time 60 min. Based on ANN data analysis, the theoretically predicted CNPs biosynthesis was 20.21 mg/mL. Under the previous conditions, the maximum experimental value of CNPs yield using *Olea* *europaea* leaves extract was 21.15 mg/mL. The theoretical predicted value of CNPs biosynthesis by ANN (20.21 mg/mL) was considerably closer to the experimental value (21.15 mg/mL), which indicates that ANN has strong prediction potential.

### In vitro effect of CNPs on biofilm formation

The current study was undertaken to examine the antibiofilm potency of biosynthesized CNPs in vitro against several MDR pathogens capable of forming biofilms, in the terms of viability, biochemical composition and hydrophobicity. Herein, *P.* *aeruginosa,* *S.* *aureus* and *C.* *albicans* were selected due to their association with nosocomial/community-acquired infections, let alone, their capability to colonize different abiotic surfaces and the cellular interfaces, resulting in major health concerns.

**Figure 10:**
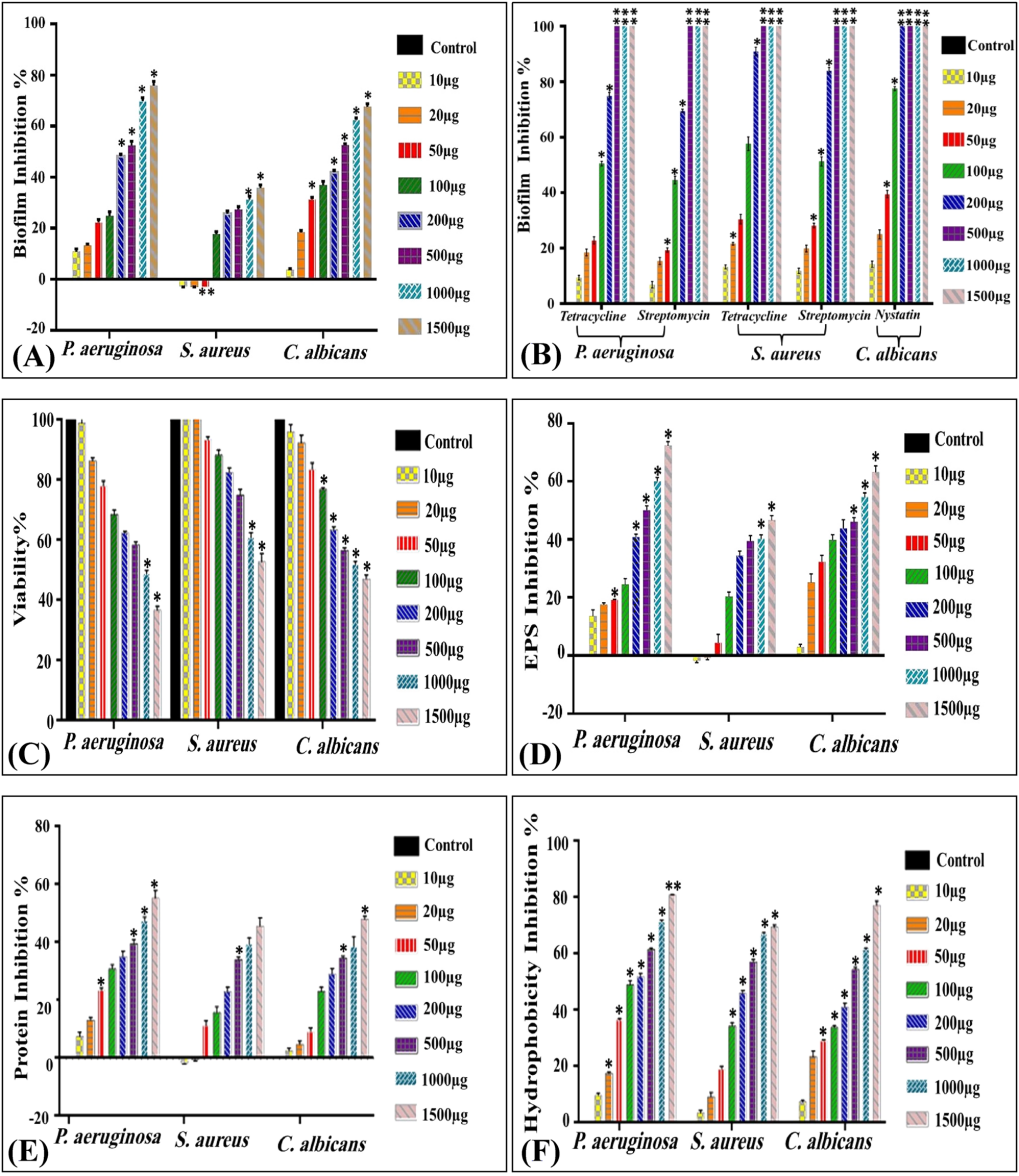
The effect of (**A**) CNPs on biofilm formation by *P.* *aeruginosa,* *S.* *aureus* and *C.* *albicans*, (**B**) different doses of standard antibiotics on biofilm formation by *P.* *aeruginosa,* *S.* *aureus* and *C.* *albicans*. CNPs, (**C**) the effect of CNPs on metabolic activity, (**D**) the effect of CNPs on EPS inhibition, (**E**) the effect of CNPs on protein inhibition, (**F**) the effect of CNPs on hydrophobicity inhibition of biofilm formation by *P.* *aeruginosa,* *S.* *aureus* and *C.* *albicans*. All values were expressed as mean ± SEM. Treatments at different concentrations were compared with control with significance of *P < 0.05.

The pattern of biofilm development exhibited significant differences (*P* ≤ 0.05) in the biofilm formation before and after treatment with CNPs. Despite higher amount of biomass formed by *P.* *aeruginosa* biofilm than that formed by *S.* *aureus,* higher inhibition percentage of biofilm formation was displayed for *P.* *aeruginosa*; revealing inter-species and even intra-species variability in their biofilm producing ability in presence of CNPs. As a general observation, among three examined pathogens, tested concentrations of antibiotics and chitosan nanoparticles progressively suppressed biofilm growth and development at elevated doses, consistent with dose-dependent biofilm inhibition. CNPs exerted different impact on biofilm formation by the pathogens examined at low concentration (10–50 μg/mL). Namely, *P.* *aeruginosa* biofilm inhibition percentages ranged from 10.88 ± 1.035 to 22.31 ± 1.19%, whereas, CNPs enhanced biofilm formation of *S.* *aureus* at exact concentrations in the range of 2.69 ± 0.255–2.925 ± 0.005%. Notably, more pronounced and significant inhibitory effect was observed upon increasing the dose to 1500 μg/mL by recording 75.96 ± 1.6 and 35.81 ± 1.19% for *P.* *aeruginosa* and *S.* *aureus* biofilms, respectively Fig. 10A. This is in agreement with Choi et al.^99^, who also found variation in *S.* *aureus,* *P.* *aeruginosa*, and *L.* *monocytogenes* biofilms formation and attributed that to physiological differences among different microbial species. In comparison, a complete inhibition for biofilm formation was recorded at higher concentrations of standard antibiotics (500, 1000 and 1500 μg/mL). Notably, at 200 μg/mL of tetracycline and streptomycin the biofilm formation was inhibited by 74.9 ± 1.2 and 90.9 ± 1.56% for *P.* *aeruginosa* and 69.5 ± 0.62 and 83.9 ± 1.14% for *S.* *aureus* (Fig. 10B). Interestingly, CNPs induced significant biofilm reduction of *C.* *albicans* biofilm in the range of 31.35 ± 0.83–67.86 ± 1.19% corresponding to concentration range of 50 -1500 μg/mL of CNPs, reflecting potent fungicidal activity. In comparison, nystatin as mycocidal agent prevented biofilm formation of *C.* *albicans* in the range of 14.2 ± 1.12–77.5 ± 0.71% at concentrations range 10–100 μg/mL and entirely suppressed biofilm formation at concentrations ranged from 200 to 1500 μg/mL.

### Effect of CNPs on biofilm metabolic activity

CV is known to bind to the entire biofilm biomass, encompassing polysaccharides in the slimy matrix and negatively charged molecules that are distributed evenly on the surface of both live and dead cells. Hence, different tetrazolium-based dyes were recruited in assessing biofilm viability in parallel to other assays such as CV assay and microscale analysis^97,98,100^.

Generally, there were common features shared between biofilm formation and viability as depicted in Fig. 10C. Notably, CNPs adversely influenced *P.* *aeruginosa* cells within the biofilm more than cells of *S.* *aureus,* in a linear dose-dependent manner. That was clearly evident through high reduction percentage of viable cells that ranged from 21.8 ± 1.175% to 63.1 ± 1.25% and 6.44 ± 0.56 to 47.11 ± 2.75% for *P.* *aeruginosa* and *S.* *aureus*, respectively at concentration range of 50–1500 μg/mL. However, low concentrations (10 and 20 μg/mL) of CNPs enhanced the viability of *S.* *aureus* cells by 3.3 ± 0.475 and 0.44 ± 0.91%, respectively. In the case of *C.* *albicans*, significant reduction (*P* ≤ 0.05) in fungal metabolic activity was obvious at 31.4 ± 0.355 to 52.76 ± 1.15%, in the concentration range of 100–1500 μg/mL. It is intuitive to infer that respiratory metabolic activity of all examined pathogens correlated significantly with biofilm biomass (r ≥ 0.9, *P* = 0.00), reflecting inhibitory effect of CNPs on active cells, whatever their distribution in multilayer architecture of biofilm. However, as previously highlighted by Silva-Dias et al.^101^, the cells at the top layer of biofilm seemed to be more active than those embedded in the basal layers.

### Effect of CNPs on biochemical composition of the biofilm

Chemical composition of the biofilm (polysaccharide, protein, e-DNA, etc.) varies for each organism and can be impacted by environmental factors^102^. These specific contents govern biofilm stability/morphology, maintain biofilm integrity, mediate cell–cell communication, facilitate cell colonization/adherence, provide nutrients and protect cells from adverse circumstances^103,104^.

The analysis of phenol–sulfuric acid content indicated a significant reduction in carbohydrate content produced by biofilms of *P.* *aeruginosa*, *S.* *aureus* and *C.* *albicans*. the content was 5.13 ± 0.035, 9.537 ± 0.01 and 6.083 ± 0.14 mg/mL in untreated control samples and reduced to 1.417 ± 0.076, 5.086 ± 0.155 and 2.238 ± 0.013 mg/mL upon CNPs treatment of *P.* *aeruginosa*, *S.* *aureus* and *C.* *albicans* biofilms, respectively, using the highest concentration 1500 µg/mL. Wherein, the inhibition percentage recorded72.39 ± 1.4, 46.67 ± 1.65 and 63.21 ± 2.13%, respectively. In the same context, 1500 µg/mL of CNPs significantly (*P* value ˂ 0.05) reduced protein content of *P.* *aeruginosa*, *S.* *aureus* and *C.* *albicans,* where protein concentration dropped from 9.68 ± 0.56, 10.97 ± 0.95 and 9.29 ± 0.64 mg/mL to 4.32 ± 0.24, 5.95 ± 0.31 and 4.93 ± 0.11 mg/mL by attaining inhibition percentages recorded 55.40 ± 2.135%, 45.75 ± 2.83% and 48.07 ± 1.15%, respectively. There appears to be a positive correlation between antibiofilm potency (Fig. 10C) and the reduction of metabolic activity, EPS (Fig. 10D) and protein content (Fig. 10E), which leads to biofilm destabilization and enfeeblement.

### Effect of CNPs on biofilm hydrophobicity

The capacity of microbial cells to colonize any surface in the form of biofilms relies on their adhesion propensity^99^. Cell surface characteristics including extracellular polymers and surface hydrophobicity are conclusive factors that facilitate microbial cell adhesion^99^. In our study, the hydrophobicity for *P.* *aeruginosa,* *S.* *aureus* and *C.* *albicans* recorded 75.3 ± 1.3, 71.0 ± 2.1 and 68.6 ± 1.7%, respectively, reflecting strong hydrophobicity property of both *P.* *aeruginosa* and *S.* *aureus*, while moderate hydrophobicity for *C.* *albicans*, as calculated by hydrophobicity index. Upon treatment with different doses of CNPs, significant reduction (*P* < 0.05) in the hydrophobicity was observed in concentration range of 100–1500 μg/mL (Fig. 10F). The cells of *P.* *aeruginosa* and *C.* *albicans* showed the highest hydrophilic index at concentration of 1500 μg/mL by recording 14.35 ± 2.3 and 15.5 ± 0.9% HI, whereas the reduction in hydrophobicity reached 80.9 ± 2.3 and 77.32 ± 3.1%, respectively comparing to the untreated control. On the other hand, the hydrophobicity index of *S.* *aureus* was 22.67 ± 3.1%; pointing out to the transition of hydrophobicity from strong phase to moderate phase at the concentration of 1500 μg/mL. In general, despite obvious structural differences in cell surfaces of the microbes examined in this study, CNPs proved effective in lowering hydrophobicity.

**Figure 11:**
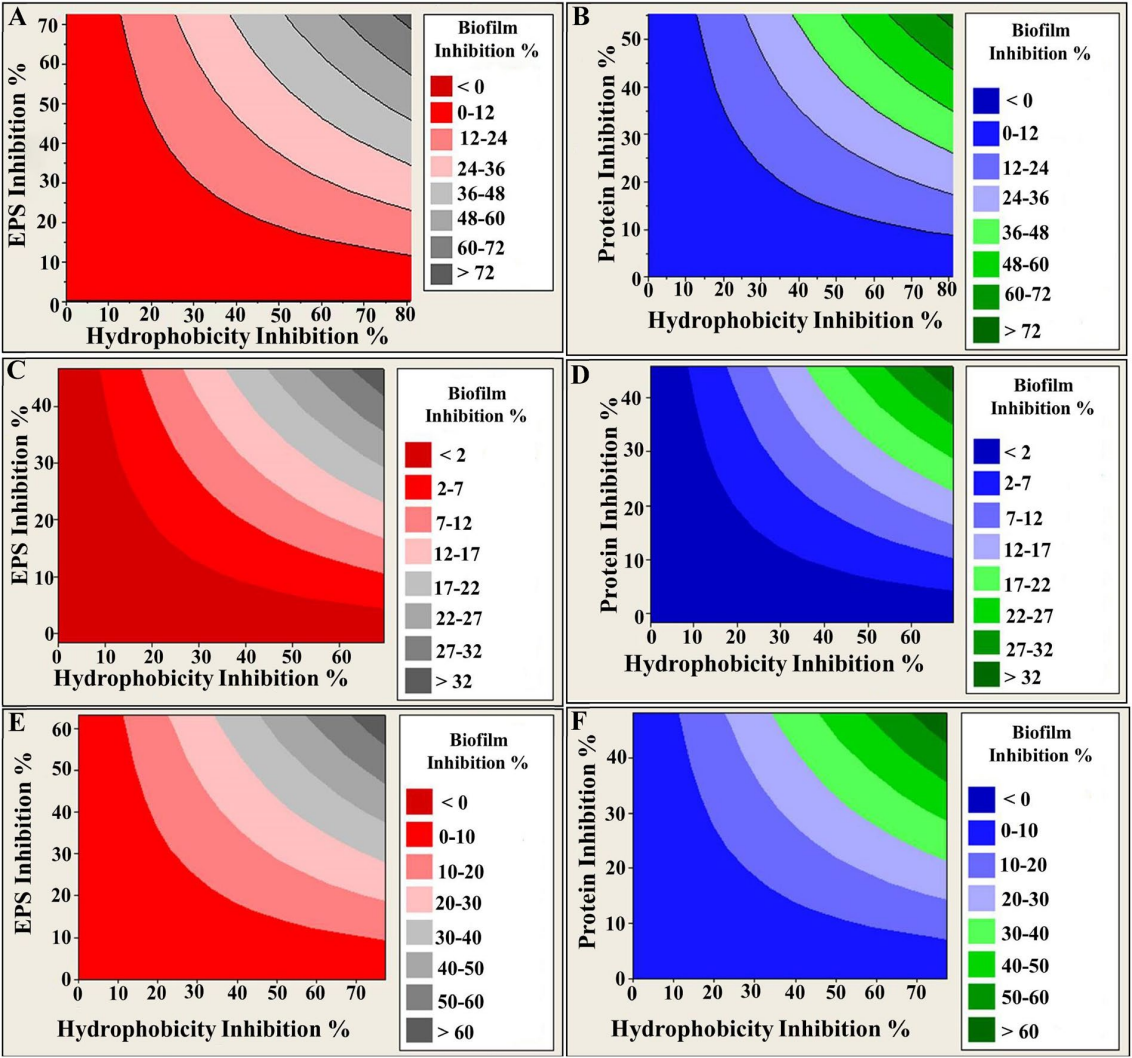
Contour plot showing the correlation of biofilm inhibition % versus EPS inhibition % (left panel), protein inhibition % (right panel) and cell surface hydrophobicity inhibition %. The graphs were plotted using Minitab 14 software. Different colors represent different levels of biofilm inhibition. (**A,B**) *P.* *aeruginosa*; (**C,D**) *S.* *aureus* and (**E,F**) *C.* *albicans.*

Interestingly, the data present here demonstrates the presence of consistent and significant correlation between the biofilm formation and EPS and protein content, and hydrophobicity as evident by Pearson’s coefficient (r ˃ 0.92, *P* = 0.00), suggesting that the modulation of biofilm development, can be exerted through influencing the microbial-surface interactions, especially by hydrophobic interactions. That would be could occur via alterations in surface-associated proteins and exopolysaccharides. It is noteworthy that hydrophobic amino acid residues of appendages such as fimbriae, pilli, or extracellular fibrils promote hydrophobicity by facilitating long-range noncovalent attachment and adherence of the cells to biological surfaces^99,105^. Additionally, exopolysaccharides, with both hydrophobic and hydrophilic components, are responsible for irreversible adhesion and the protection of the growing cells within the biofilm^106,107^. Caccavo et al.^108^, stated that cells within the biofilm with predominant exopolysaccharide content showed significantly less hydrophobicity than those with higher protein content. Here we show the graphical representations for the correlation between biofilm formation, hydrophobicity and ePSs/protein were depicted via contour plots (Fig. 11). This is in agreement with Pompilio et al.^109^, who found that the biofilm of *Stenotrophomonas* *maltophilia* displayed higher hydrophobicity once its cells attached to the substratum and attributed this correlation to exopolysaccharide content on the cell surface.

Based on the preceding analytical data, the antagonistic mechanism of CNPs could be proposed, which intrinsically lies behind their nature and size. The polycationic nature of chitosan can be enhanced by the functional amino groups (NH^3+^) of glucosamine units, particularly under neutral or alkaline conditions, allows enhanced electrostatic binding with negatively charged residues scattered on microbial cell membranes^110^. The data presented here indicate that chitosan molecules are more effective in treating gram-negative bacteria than gram-positive, possibly due to greater distribution of phosphate and pyrophosphate functional groups on lipopolysaccharides and phospholipids on the outer membrane of gram-negative bacteria. These contribute the cells negative charge and are able to adsorb more cationic charges, allowing for more lethal impact of chitosan. Let alone higher hydrophobicity of gram-negative bacteria may also contribute to their susceptibility. In contrast, lower hydrophobicity and thick peptidoglycan layer with low density of negative charge render gram-positive bacteria less susceptible to chitosan. Nevertheless, chitosan effectiveness against both bacterial groups had been controversial, with some investigations reporting stronger effects of chitosan against gram-positive bacteria than gram-negative bacteria^111^, and others documenting the fungistatic activity of chitosan rather than fungicidal, ascribing this to morphological alterations in cell wall^110^. Interestingly, our results contradict those studies. Promising mycocidal potency was observed by CNPs in treating *C.* *albicans*. Such efficacy could be related to the prevalence of negatively charged unsaturated fatty acids on the fungal cell surface, especially for *Candida* spp, which boost membrane fluidity, and therefore, allows close interaction with CNPs. In addition, their influence on fungal mitochondrial function was reported^112^. The results of the current study, however, suggest that CNPs could, potentially, be used to treat candidiasis among immunocompromised patients, particularly those admitted to intensive care units (ICUs) with secondary pneumonia after COVID-19 infection.

It is noteworthy that our overall results follow linear dose-dependent manner, which could be attributed to the availably of cationic sites that tend to aggregate in the form of sheath or coat, which surround microbial cells, preventing by such way nutrients penetration and efflux of metabolic byproducts externally^113^. Hence, based on their natural characteristics -alone, chitosan molecules, as a natural polymer, could be described as external outer membrane disruptor in lieu of internal penetrator^110^. However, due to the small size range of CNPs (6.91 to 11.14 nm) which were associated with the higher surface area ratio, its ability to diffuse into the biofilm structure or into cells through porin channels, increases its potency and more microbial damage occurs easily^114^.

The antibiofilm activity of CNPs could be seen in all phases of the multi-step process of biofilm formation. Initially, in planktonic or free-floating stage, the CNPs bind tightly to microbial cell surface, leading to cell membrane destabilization, hydrolysis of the peptidoglycans, ruining of microbial adhesive structure, reducing dehydration of cell surface, dampening of interactions (van der Walls, electrostatic, hydrophobic) between cells and the substrate (biotic or abiotic); eventually hindering aggregation or attachment step^114^. Other lethal effects extend further and include wall permeability, osmotic imbalances, intracellular leakage of electrolytes and proteinaceous constituents. In the second stage of biofilm formation, the “cell adhesion”, which is characterized by production of extracellular polymeric matrix that binds cell monolayers and microcolonies in an irreversible attachment, CNPs prohibit exopolysaccharide production, disrupt the formed polysaccharide backbone and decrease hydrophobicity. Meanwhile, they also interact with proteinaceous moieties, RNA and eDNA, causing denaturation and inactivation, blocking quorum sensing receptors (cell to cell communication strategy), and ultimately disrupt microbial colonization, irreversible adhesion and entire biofilm development^114^.

Finally, CNPs exhibited destructive capability against biofilm architecture with all constituents (metabolic active cells, protein and carbohydrate moieties), which encourage their recruitment as an effective environmentally friendly alternative anti-biofoulant or anti-adhesion coating agent, to known organic antifoulants such as s-triazine Irgarol 1051, Sea-nine 211, zinc pyrithione or synthetic chemicals as chalcone derivatives which are hazardous for aquatic ecosystems and threaten aquatic fauna and flora^105^. Moreover, as a natural, biosafe and biocompatible polymer in nanoform with outstanding biological activity, CNPs could be harnessed in adjuvant therapy and prophylactic means.

## Data Availability

All data generated or analyzed during this study are included in this article.
